# Long-term outcomes of bilateral salpingo-oophorectomy in women with personal history of breast cancer

**DOI:** 10.1136/bmjonc-2024-000574

**Published:** 2025-02-25

**Authors:** Hend Hassan, Tameera Rahman, Andrew Bacon, Craig Knott, Isaac Allen, Catherine Huntley, Lucy Loong, Yvonne Walburga, Eva Morris, Steven Hardy, Bethany Torr, Diana M Eccles, Clare Turnbull, Marc Tischkowitz, Paul Pharoah, Antonis C Antoniou

**Affiliations:** 1 Centre for Cancer Genetic Epidemiology, University of Cambridge, Cambridge, UK; 2 National Disease Registration Service, London, London, UK; 3 Health Data Insight CIC, Cambridge, UK; 4 Division of Genetics and Epidemiology, The Institute of Cancer Research, London, UK; 5 Big Data Institute, University of Oxford, Oxford, UK; 6 University of Southampton, Southampton, UK; 7 Department of Medical Genetics, University of Cambridge, Cambridge, Cambridgeshire, UK; 8 Cedars-Sinai Medical Center, Los Angeles, California, USA

**Keywords:** Breast cancer (female), Ovarian cancer, Epidemiology, Surgical oncology

## Abstract

**Objectives:**

To investigate the association between bilateral salpingo-oophorectomy (BSO) and long-term health outcomes in women with a personal history of breast cancer.

**Methods and analysis:**

We used data on women diagnosed with invasive breast cancer between 1995 and 2019 from the National Cancer Registration Dataset (NCRD) in England. The data were linked to the Hospital Episode Statistics-Admitted Patient Care dataset to identify BSO delivery. Long-term health outcomes were selected from both datasets. Multivariable Cox regression was used to examine the associations, with BSO modelled as a time-dependent covariate. The associations were investigated separately by age at BSO.

**Results:**

We identified 568 883 women, 23 401 of whom had BSO after the breast cancer diagnosis. There was an increased risk of total cardiovascular diseases with an HR of 1.10 (95% CI 1.04 to 1.16) in women who had BSO<55 years and 1.07 (95% CI 1.01 to 1.13) for women who had BSO≥55 years. There was an increased risk of ischaemic heart diseases, but there was no association with cerebrovascular diseases. BSO at any age was associated with an increased risk of depression (HR 1.20, 95% CI 1.12 to 1.28) and increased risk of second non-breast cancer in older women (HR 1.21, 95%CI 1.08 to 1.35). BSO in older women was associated with reduced risk of all-cause mortality (HR 0.92, 95% CI 0.87 to 096), but not in women who had BSO<55 years.

**Conclusion:**

In women with a personal history of breast cancer, BSO before and after the age of 55 years is associated with an increased risk of long-term outcomes. BSO after 55 years is associated with reduced all-cause mortality. Family history or genetic predisposition may confound these associations.

**WHAT IS ALREADY KNOWN ON THIS TOPIC**

- Prior research has shown that bilateral salpingo-oophorectomy (BSO) is associated with a reduction in ovarian cancer risk but an increase in the risk of adverse long-term health outcomes. Though, there is a gap in examining the long-term outcomes of BSO in women with a personal history of breast cancer.

**WHAT THIS STUDY ADDS**

- Our findings indicate an increased risk of total cardiovascular diseases and depression in women who underwent BSO. However, there is a reduction in all-cause mortality in women who had BSO after the age of 55 years.

**HOW THIS STUDY MIGHT AFFECT RESEARCH, PRACTICE OR POLICY**

- We hope that our study will aid in creating personalised counselling and enhancing decision-making for women with a personal history of breast cancer who opt for BSO.

## Introduction

Women with a personal history of breast cancer are at increased risk of developing second cancers including ovarian cancer.^1^ The cumulative 20-year risk of developing ovarian cancer after a breast cancer diagnosis has been estimated to be 1.4% for women diagnosed with breast cancer before the age of 50 and 1.9% for those diagnosed at the age of 50 years or older.^2^ There is no effective modality for screening for ovarian cancer. The only recommended method for prevention in women at elevated risk for ovarian cancer is bilateral salpingo-oophorectomy (BSO). BSO is associated with more than a 90% reduction in the risk of ovarian cancer.^3 4^ However, the benefit of ovarian cancer risk reduction should be balanced against the health sequelae caused by the premature loss of oestrogen. In women with no history of cancer BSO with hysterectomy for benign indications has been found to be associated with reduced risk of ovarian cancer and breast cancer and increased risk of cardiovascular diseases (CVD), depression, dementia, cancer and all-cause mortality.^5^

BSO is also performed to suppress ovarian function in women with oestrogen receptor-positive breast cancer. Ovarian function suppression (OFS) could be achieved by surgical removal of both ovaries or radiation-induced ablation both of which are permanent, or by using gonadotropin-releasing hormone agonists which results in temporary suppression of ovarian function. Oestrogen cessation might lower the risk of recurrence, contralateral breast cancer (CBC) and mortality.^6^ The present study focuses on other long-term outcomes of BSO after breast cancer diagnosis, including CVD, neuropsychiatric outcomes and second cancer occurrence. This has not been studied before at a population scale using electronic health records. Evidence is limited and focuses only on mortality outcomes^7 8^ or on women who had the BSO prior to the diagnosis of breast cancer.^9^ Also, evidence derived from the general population is often used to counsel high-risk women who are opting for BSO.

Using general population data to counsel women with a personal history of breast cancer presents challenges, as these women may exhibit different benefit-risk profile, for example, reproductive history, family history of cancer, weight and alcohol consumption.^10^ These in turn may influence their baseline risk of developing long-term health sequelae. Tumour characteristics and treatment choices could confound the association between BSO and the long-term outcomes.^11 12^ Moreover, in the general population, BSO is often performed at the time of hysterectomy which means that the evidence could be confounded by the indication for the hysterectomy or by the potential long-term outcomes of hysterectomy alone in the comparison groups used.^5^ Hence, there is a need for guidance to be based on studies specifically conducted on these women. Women should be able to make decisions based on accurate knowledge of the risks and benefits of the BSO. This information is particularly relevant in women with a personal history of breast cancer who may have additional concerns about using hormonal replacement therapy.

This study aims to investigate the association between BSO and long-term health outcomes, compared with receiving standard treatment after a breast cancer diagnosis. This is the largest study to date to examine these associations, using population-scale linked data from the National Cancer Registration Dataset (NCRD) and Hospital Episode Statistics-Admitted Patient Care (HES-APC) in England.

## Methods

### Data sources

#### National Cancer Registration Dataset

The NCRD is collected and managed by the National Cancer Registration and Analysis Service (NCRAS), a population-based cancer registry for England with national coverage since 1971.^13^ NCRAS collects data from multiple sources including multidisciplinary team meetings, pathology reports, molecular testing results, treatment records and hospital activity records. The NCRD contains demographic data including the age at diagnosis, gender, deprivation index, tumour characteristics data including the cancer stage using the TNM (tumour, node, metastasis) staging system, grade, hormonal receptor status, tumour morphology, tumour size, number of nodes excised and treatment data on the receipt of radiotherapy, chemotherapy, immunotherapy and surgery. The Office for National Statistics provides NCRAS with data on the date and cause of death. Section 254 of the Health and Social Care Act 2012 allows NCRAS to collect individual-level data on patients with cancer without consent.

#### Hospital Episode Statistics-Admitted Patient Care

HES-APC collects data on all NHS hospital admissions in England. HES-APC also collects data on admissions to independent providers funded by the NHS.^14^ The NHS funds more than 98% of the hospital activity in England.^14^ HES-APC includes all hospital care episodes since the financial year 1989/1990. Data fields include diagnoses, procedures, patient demographics and admission and discharge dates. Diagnoses are coded using the International Classification of Diseases version 10 (ICD-10). Each admission could have up to 20 diagnoses. We considered any of the first three diagnosis fields as the primary diagnosis/es and the other diagnosis fields as comorbidities. Procedures are coded using OPCS4 codes (Office of Population, Census and Surveys Classification of Interventions and Procedures, fourth Revision). Pseudonymised patient identifiers allow linkage of HES APC to NCRD.

### Inclusion and exclusion criteria

**Figure 1:**
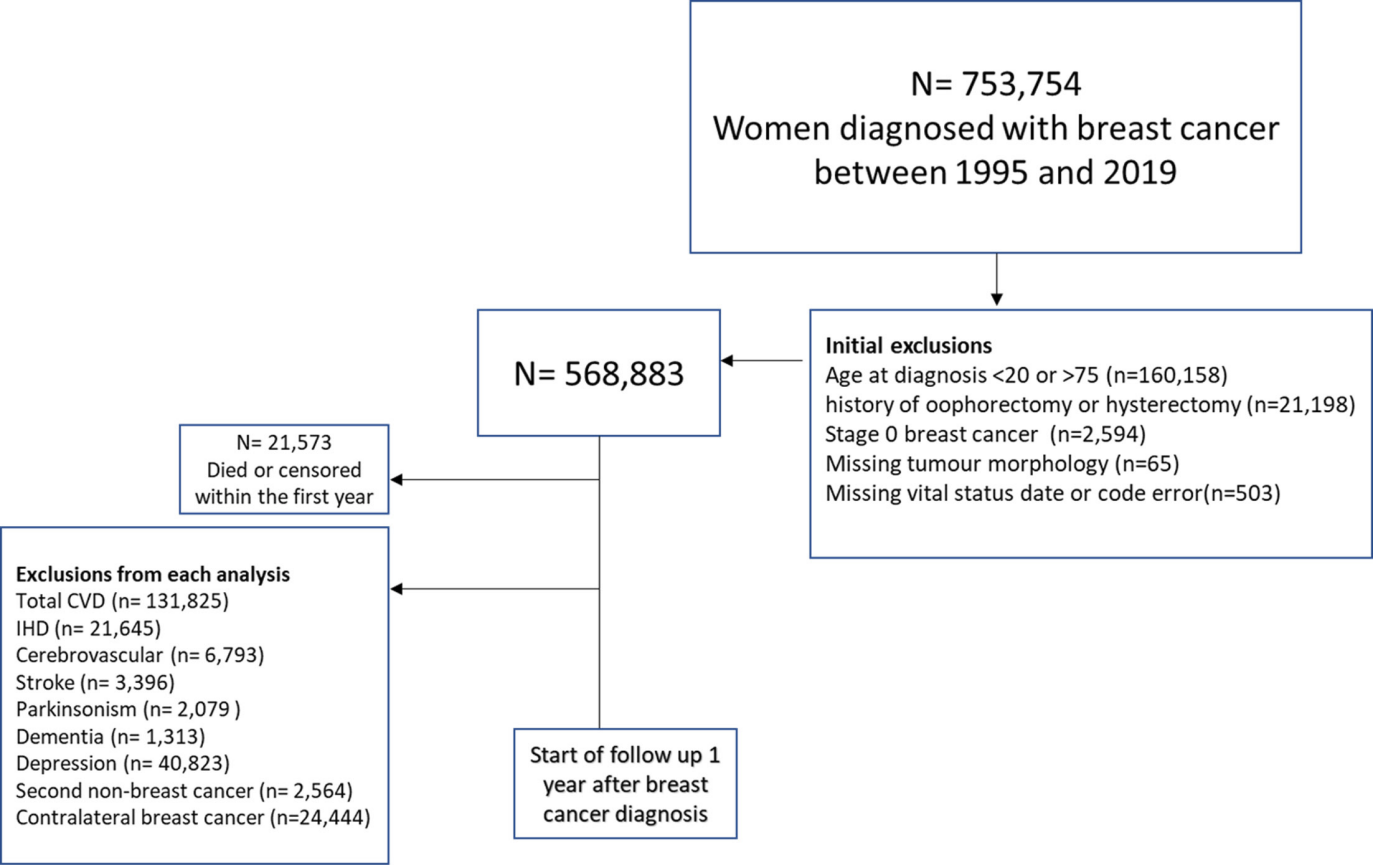
Exclusion process among women diagnosed with breast cancer between 1995 and 2019. Patients diagnosed with a particular outcome before the start of the follow-up were excluded from the analysis of that outcome. CVD: Cardiovascular diseases ; IHD: Ischaemic heart diseases.

We included women diagnosed with invasive breast cancer between 1995 and 2019, diagnosed between the ages 20 and 75 and who had no history of previous cancer diagnosis. We excluded women with a history of hysterectomy or oophorectomy before the date of breast cancer diagnosis. The number of women excluded for different reasons is summarised in figure 1. For each outcome investigated, women diagnosed with the specific outcome (in any of the 20 HES diagnosis fields) before or within the first year of the breast cancer diagnosis were excluded from the analysis. For the association with CBC, women with bilateral tumours or unknown laterality of the first tumour were excluded from the analysis.

### Identifying BSO procedures

BSO procedures were identified using the OPCS4 Q221 and Q223 codes in HES-APC. Hysterectomy procedures were identified using the codes such as Q073, Q074, Q075, Q078, Q079, Q081, Q082, Q083, Q088 and Q089. BSO was considered to be for a malignant indication if the patient was diagnosed with a gynaecological malignancy 1 year before or 1 year after the procedure date.

### Identifying long-term outcomes

Outcomes of interest were all-cause mortality, total CVD, ischaemic heart diseases (IHD), cerebrovascular diseases, dementia, depression and parkinsonism (extrapyramidal movement disorders), breast cancer mortality, non-breast cancer mortality, CBC and second non-breast cancer (excluding non-melanoma skin cancer). Cancer outcomes and mortality data were identified from the NCRD. Breast cancer-specific mortality was defined as death where breast cancer was listed as a cause on part I of the death certificate. Non-cancer outcomes were identified from HES admissions using the first three diagnosis fields (primary diagnosis) or from part Ia of the death certificate. The ICD-10 codes used are summarised in online supplemental table S1. The associations with the severity of CVD outcomes (fatal/non-fatal) were assessed separately. Fatal outcomes were outcomes identified either from the cause of death or from HES admissions followed by fatality within the first 48 hours after the admission. Non-fatal were outcomes identified from HES admissions which were not followed by death within the first 48 hours.

### Follow-up and censoring

Follow-up started 1 year after the date of breast cancer diagnosis to avoid misclassifying bilateral breast cancers as second primary cancers and to allow time for exclusion of cases with any comorbidities identified after the breast cancer diagnosis. Follow-up ended at the first of the following: the outcome of interest, censoring or end of data collection. Data collection ended on 30 December 2020 for cancer outcomes and 1 May 2022 for mortality and non-cancer outcomes. Follow-up time was censored at the time of unilateral oophorectomy, pelvic clearance or bilateral oophorectomy for malignant indications (except for ovarian cancer and second non-breast cancers analyses). In addition, for the CBC analysis women were censored 1 year after a breast surgery on the opposite side or a breast surgery with unknown laterality occurring more than 1 year after the breast cancer diagnosis. In each separate analysis for an outcome of interest, women who developed any of the other outcomes studied here were still followed for the outcomes of interest. Follow-up for BSO started 1 year after the surgery date, to allow time for biological plausibility (allow time for the cessation of oestrogen to cause a pathological effect) and to minimise detection bias. We performed separate analyses by the age at surgery, BSO<55 years in younger women and BSO≥55 years in older women. The age 55 was chosen as a proxy for the age at menopause. According to the British Menopause Society, it is estimated that more than 80% of women will be menopausal by the age of 54.^15^ In the younger women analysis, we only included women diagnosed with breast cancer before the age of 55. Women who were diagnosed before age 55 and had BSO after age of 55 years were included in the non-BSO group and censored at the date of their surgery, the occurrence of events, whichever occurred first.

### Multiple imputation of missing data

We used multivariate imputations by chained equations to impute the missing TNM stage, grade, oestrogen receptor (ER) status, human epidermal growth factor receptor 2 (HER2) status, tumour size, number of lymph nodes excised, ethnicity and Charlson Comorbidity Index (online supplemental materials).

### Statistical analysis

We used Cox regression to calculate HRs for the association between BSO and the long-term outcomes, with BSO modelled as a time-dependent covariate. The models were adjusted for age at breast cancer diagnosis, year of diagnosis, tumour size, number of excised lymph nodes, M-stage, grade, ER status, HER2 status, ethnicity (white, Asian, black, mixed and other), deprivation index (1-least deprived, 2, 3, 4 and 5-most deprived) and Charlson Comorbidity Index. The Charlson Comorbidity Index is a weighted scale that predicts the risk of mortality within 1 year of hospitalisation.^16^ The index was derived from HES-APC records for all the patients 6 years prior to the breast cancer diagnosis and calculated using the method described by Quan *et al*.^17^ The association with second cancer was further adjusted for hysterectomy. A regression model was fitted using each of the imputed datasets and the log (HRs) were combined using Rubin’s rule.^18^ We fitted the models for the CBC and second non-breast cancer analyses with an interaction term between BSO and M-stage (0/1). This was done to provide estimates for the association in women with M-stage 0. The Cox-regression proportional hazard assumption was assessed by plotting the Schoenfeld residuals (supplementary materials 2: online supplemental figure 2S–17S). All analyses were carried out using R (V.4.1.0; R Foundation for Statistical Computing, Vienna, Austria).

### Sensitivity analyses

We assessed whether the findings were influenced after adjustment for hysterectomy. We also examined the associations between the long-term outcome and three types of procedures (hysterectomy alone, hysterectomy and BSO, BSO alone).

We conducted sensitivity analyses for the associations between the long-term outcomes and BSO in older women by restricting the analyses to women diagnosed with breast cancer at age 55 years or older.

To examine whether the association with breast cancer mortality is explained by CBC diagnosis, we conducted a sensitivity analysis where CBC diagnosis served as a censoring event. Given that the initial cohort analysis for assessing the association with breast cancer mortality included women with bilateral tumours or unknown literalities of the first tumour, we repeated the analysis after excluding women with unknown first tumour literalities or bilateral tumours. This was done while retaining the initial censoring methodology, and subsequently employing CBC as a censoring event to allow comparability of the results.

### Patients and public involvement

The CanGene-CanVare programme includes a Patient Reference Panel (PRP) made up of patients, carers and members of the public who are, or have been, affected by cancer. The PRP is involved in programme governance and oversight, contributing personal perspectives and experiences as well as in the communication and dissemination of programme output.

## Results

We identified 568 883 women, 23 401 of whom had BSO after the breast cancer diagnosis, 8243 after the age of 55 years and 15 158 before the age of 55 years. Baseline demographic, tumour and clinical characteristics of women who had BSO (<55 and ≥55 years) alongside their corresponding reference groups are shown in table 1. The median age at diagnosis for women who had BSO<55 years was 43 years (IQR 38–47 years) and for women in the reference group was 48 years (IQR 43–51 years). The median age at diagnosis in women who had BSO≥55 years was 57 years (IQR:52–63 years) and for women in the corresponding reference group 58 years (IQR 50–66). The percentage of white women in the BSO groups was higher than in the non-BSO groups, in the ‘≥55 years’ BSO group 94% were white versus 86% in the reference group and in the ‘<55 years’ BSO group 92% were white versus 84% in their reference group. Among the women who had BSO≥55 years 65% had hysterectomy compared with 2% in the reference group and 43% in those who had BSO<55 years compared with 3% in the reference group.

**Table 1:** Baseline characteristics of women who had BSO above or after the age of 55 with their respective reference groups used in the association analyses

Bilateral salpingo-oophorectomy	YesAge at BSO<55n**=**15 158	NoReference*n=221 892	YesAge at BSO≥55n=8243	NoReference**†****n=**545 482
Diagnosis year				
Median (IQR)	2010 (2004–2014)	2009 (2002–2015)	2006 (2000–2012)	2009 (2003–2015)
Age at diagnosis				
Median (IQR)	43 (38–47)	48 (43–51)	57 (52–63)	58 (50–66)
Age at BSO				
Median (IQR)	47 (43–50)	57 (56–61) ‡	63 (58–70)	
Follow-up years				
Median (IQR)	11 (7–17)	10 (6–17)	14 (9–20)	9 (5–15)
Deprivation index				
1—least deprived	3491 (23%)	52 702 (24%)	2009 (24%)	128 066 (23%)
2	3515 (23%)	50 084 (23%)	2120 (26%)	125 329 (23%)
3	3190 (21%)	45 113 (20%)	1736 (21%)	113 116 (21%)
4	2700 (18%)	39 413 (18%)	1395 (17%)	96 451 (18%)
5—most deprived	2262 (15%)	34 580 (16%)	983 (12%)	82 520 (15%)
Ethnicity				
White	13 988 (92%)	186 939 (84%)	7721 (94%)	471 331 (86%)
Asian	375 (2.5%)	8184 (3.7%)	125 (1.5%)	14 913 (2.7%)
Black	199 (1.3%)	4566 (2.1%)	48 (0.6%)	7300 (1.3%)
Mixed	100 (0.7%)	1416 (0.6%)	25 (0.3%)	2240 (0.4%)
Other	163 (1.1%)	3065 (1.4%)	41 (0.5%)	5610 (1.0%)
Missing	333 (2.2%)	17 722 (8.0%)	283 (3.4%)	44 088 (8.1%)
TNM stage				
1	4259 (28%)	62 373 (28%)	3063 (37%)	180 007 (33%)
2	5242 (35%)	69 784 (31%)	2070 (25%)	154 457 (28%)
3	1331 (8.8%)	16 243 (7.3%)	329 (4.0%)	33 840 (6.2%)
4	315 (2.1%)	6104 (2.8%)	90 (1.1%)	17 064 (3.1%)
Missing	4011 (26%)	67 388 (30%)	2691 (33%)	160 114 (29%)
Grade				
1	1649 (11%)	31 674 (14%)	1633 (20%)	86 678 (16%)
2	6673 (44%)	92 744 (42%)	3787 (46%)	245 097 (45%)
3	5983 (39%)	79 652 (36%)	2217 (27%)	166 880 (31%)
Missing	853 (5.6%)	17 822 (8.0%)	606 (7.4%)	46 827 (8.6%)
ER status				
Positive	6527 (43%)	79 593 (36%)	2687 (33%)	206 476 (38%)
Negative	984 (6.5%)	18 067 (8.1%)	428 (5.2%)	39 371 (7.2%)
Missing	7647 (50%)	124 232 (56%)	5128 (62%)	299 635 (55%)
HER2 status				
Positive	1109 (7.3%)	17 333 (7.8%)	287 (3.5%)	36 024 (6.6%)
Negative	6251 (41%)	80 291 (36%)	2470 (30%)	206 536 (38%)
Missing	7798 (51%)	124 268 (56%)	5486 (67%)	302 922 (56%)
Morphology				
Invasive ductal carcinoma	12 191 (80%)	174 050 (78%)	6075 (74%)	410 727 (75%)
Invasive lobular carcinoma	1457 (9.6%)	20 964 (9.4%)	1051 (13%)	62 170 (11%)
Other	1510 (10%)	26 878 (12%)	822 (14%)	56 611 (13%)
Death events				
No	12 576 (83%)	165 405 (75%)	6205 (75%)	357 566 (66%)
Yes	2582 (17%)	56 487 (25%)	2038 (25%)	187 916 (34%)
Hormonal treatment				
No	9761 (64%)	149 666 (67%)	4890 (59%)	339 772 (62%)
Yes	5397 (36%)	72 226 (33%)	3353 (41%)	205 710 (38%)
Radiotherapy treatment				
No	5517 (36%)	81 066 (37%)	3162 (38%)	207 378 (38%)
Yes	9641 (64%)	140 826 (63%)	5081 (62%)	338 104 (62%)
Chemotherapy				
No	6061 (40%)	106 622 (48%)	5697 (69%)	346 938 (64%)
Yes	9097 (60%)	115 270 (52%)	2546 (31%)	198 544 (36%)
Hysterectomy				
Yes	6478 (43%)	6439 (2.9%)	5321 (65%)	11 419 (2.1%)
No	8680 (57%)	215 453 (97%)	2922 (35%)	534 063 (98%)

* Women who were diagnosed with invasive breast cancer before the age of 55 and did not have a BSO before the age of 55.

† Women who were diagnosed with invasive breast cancer at any age and did not have a BSO.

‡ Reference includes women who were diagnosed before the age of 55 and had a BSO after the age of 55, these women were censored at the date of their BSO≥55, outcome development or occurrence of a censoring event whichever occurred first.

BSObilateral salpingo-oophorectomyER, oestrogen receptor; HER2, human epidermal growth factor receptor 2TNMstage, tumour, node, metastasis stage

Summaries of the numbers at risk, person years and number of events in the cohort and the HRs for the associations between BSO and the long-term outcomes are shown in table 2.

**Table 2:** Associations between BSO and long-term outcomes by age at BSO

Outcome		Age at BSO	N at risk	N events	Person years	HR (95% CI)*
All-cause mortality		Unstratified	547 246	158 447	5 108 170	1.01 (0.98 to 1.05)
		<55	231 281	50 053	2 304 019	1.03 (0.98 to 1.08)
		≥55	532 118	156 083	4 947 996	0.92 (0.87 to 0.96)
Total CVD	Total	Unstratified	415 485	104 491	3 570 242	1.09 (1.05 to 1.13)
	Non-fatal			100 279	3 570 245	1.10 (1.05 to 1.14)
	Fatal			11 546	4 157 063	0.93 (0.82 to 1.06)
	Total	<55	206 942	33 189	1 935 037	1.10 (1.04 to 1.16)
	Non-fatal			32 070	1 935 038	1.10 (1.05 to 1.16)
	Fatal			2153	2 119 670	0.88 (0.70 to 1.11)
	Total	≥55	401 756	102 312	3 434 178	1.07 (1.01 to 1.13)
	Non-fatal			98 144	3 434 181	1.08 (1.02 to 1.15)
	Fatal			11 461	4 008 443	0.88 (0.75 to 1.03)
IHD	Total	Unstratified	525 665	28 178	4 805 042	1.15 (1.06 to 1.24)
	Non-fatal			25 710	4 805 043	1.17 (1.08 to 1.27)
	Fatal			3549	4 951 005	0.81 (0.61 to 1.06)
	Total	<55	229 018	5871	2 250 590	1.20 (1.06 to 1.37)
	Non-fatal			5556	2 250 590	1.21 (1.07 to 1.38)
	Fatal			428	2 285 240	0.93 (0.53 to 1.63)
	Total	≥55	510 653	27 873	4 647 631	1.15 (1.05 to 1.27)
	Non-fatal			25 418	4 647 632	1.20 (1.08 to 1.32)
	Fatal			3534	4 791 818	0.78 (0.56 to 1.06)
Cerebrovascular diseases	Total	Unstratified	540 517	20 141	5 002 065	1.00 (0.91 to 1.11)
	Non-fatal			18 203	5 002 068	1.01 (0.91 to 1.12)
	Fatal			4106	5 068 176	0.93 (0.73 to 1.18)
	Total	<55	230 308	3512	2 282 799	0.92 (0.77 to 1.10)
	Non-fatal			3207	2 282 800	0.94 (0.78 to 1.12)
	Fatal			518	2 296 864	0.94 (0.59 to 1.48)
	Total	≥55	525 428	19 980	4 842 903	0.98 (0.87 to 1.10)
	Non-fatal			18 052	4 842 906	1.00 (0.88 to 1.13)
	Fatal			4085	4 908 357	0.83 (0.62 to 1.10)
Angina		Unstratified	525 664	11 760	4 872 452	1.27 (1.13 to 1.42)
		<55	229 017	2740	2 265 645	1.28 (1.06 to 1.54)
		≥55	510 652	11 609	4 714 269	1.34 (1.16 to 1.54)
Myocardial infarction		Unstratified	525 664	9074	4 917 312	1.03 (0.89 to 1.18)
		<55	229 017	1747	2 277 193	0.84 (0.65 to 1.09)
		≥55	510 652	9004	4 758 434	1.09 (0.92 to 1.29)
Chronic IHD		Unstratified	525 664	19 686	4 849 348	1.08 (0.99 to 1.19)
		<55	229 017	4033	2 262 022	1.14 (0.98 to 1.34)
		≥55	510 652	19 488	4 691 219	1.12 (1.00 to 1.25)
Haemorrhagic stroke		Unstratified	543 914	4584	5 076 992	0.97 (0.80 to 1.19)
		<55	230 728	1101	2 296 233	0.96 (0.71 to 1.29)
		≥55	528 808	4530	4 917 201	0.90 (0.69 to 1.18)
Ischaemic stroke		Unstratified	543 914	9971	5 055 430	1.04 (0.90 to 1.19)
		<55	230 728	1513	2 294 263	0.92 (0.70 to 1.20)
		≥55	528 808	9902	4 895 713	1.06 (0.90 to 1.24)
Parkinsonism		Unstratified	545 231	3533	5 082 902	0.78 (0.61 to 1.01)
		<55	230 926	648	2 298 880	0.98 (0.67 to 1.44)
		≥55	530 135	3493	4 923 142	0.67 (0.48 to 0.94)
Dementia		Unstratified	545 933	12 036	5 072 461	0.94 (0.81 to 1.08)
		<55	231 212	625	2 301 234	0.90 (0.55 to 1.47)
		≥55	530 804	12 016	4 912 385	0.95 (0.82 to 1.11)
Depression		Unstratified	506 423	21 243	4 784 915	1.20 (1.12 to 1.28)
		<55	213 588	10 491	2 150 730	1.18 (1.09 to 1.28)
		≥55	492 741	20 103	4 640 259	1.18 (1.05 to 1.33)
Second non-breast cancer†		Unstratified	544 682	39 886	4 494 403	1.05 (0.97 to 1.14)
		<55	230 904	11 049	2 044 405	1.03 (0.91 to 1.17)
		≥55	529 581	39 362	4 353 510	1.21 (1.08 to 1.35)
Contralateral breast cancer		Unstratified	522 802	13 948	4 071 952	1.04 (0.95 to 1.15)
		<55	221 647	6819	1 799 643	1.01 (0.89 to 1.14)
		≥55	508 460	13 179	3 967 272	1.13 (0.97 to 1.33)
Breast cancer mortality		Unstratified	547 246	80 795	5 107 400	1.11 (1.06 to 1.16)
		<55	231 281	36 745	2 303 692	1.09 (1.04 to 1.15)
		≥55	532 118	78 865	4 947 241	0.96 (0.89 to 1.05)
Non-breast cancer mortality		Unstratified	547 246	77 629	5 107 400	0.91 (0.86 to 0.96)
		<55	231 281	13 300	2 303 692	0.86 (0.78 to 0.96)
		≥55	532 118	77 196	4 947 241	0.93 (0.88 to 0.99)

* Additionally adjusted for hysterectomy.Models adjusted for age at breast cancer diagnosis, year of diagnosis, tumour size, number of excised lymph nodes, M-stage, grade, ER status, HER2 status, ethnicity, deprivation index and Charlson Comorbidity Index.

† Models adjusted for age at breast cancer diagnosis, year of diagnosis, tumour size, number of excised lymph nodes, M-stage, grade, ER status, HER2 status, ethnicity, deprivation index and Charlson comorbidity indexAdditionally adjusted for hysterectomy.

BSObilateral salpingo-oophorectomyCVD, cardiovascular diseases; ERoestrogen receptorHER2human epidermal growth factor receptor 2IHD, ischaemic heart diseasesN, number

### Associations with cardiovascular outcomes

There was an increased risk of total CVD with an HR of 1.10 (95% CI 1.04 to 1.16) in women who had BSO<55 years and an HR of 1.07 (95% CI 1.01 to 1.13) for women who had BSO≥55 years. There was an increased risk of IHD for both younger and older women with HRs of 1.20 (95% CI 1.06 to 1.37) and 1.15 (95% CI 1.05 to 1.27), respectively. Further exploration of associations with IHD subtypes, BSO was significantly associated only with angina (unstratified HR 1.27, 95% 1.13 to 1.42), but not with myocardial infarction (unstratified HR 1.03, 95% CI 0.89 to 1.18) or chronic IHD (unstratified HR 1.08, 95% CI 0.99 to 1.19).

There was no association between BSO and cerebrovascular diseases, haemorrhagic stroke or ischaemic stroke with unstratified HR estimates 1.00 (95% CI 0.91 to 1.11), 0.97 (95% CI 0.80 to 1.19) and 1.04 (95% CI 0.90 to 1.19), respectively. Investigating the associations by the severity of the CVD outcomes yielded significant associations only with the non-fatal total CVD and non-fatal IHD outcomes.

### Association with neuropsychiatric outcomes

BSO was associated with an increased risk of depression both in women who had BSO<55 y (HR 1.18, 95% CI 1.09 to 1.28) and ≥55 years (HR 1.18, 95% CI 1.05 to 1.33). BSO was not associated with parkinsonism in women who had BSO<55 years (HR 0.98, 95% CI 0.67 to 1.44), but was associated with a reduced risk of parkinsonism in those who had BSO≥55 years (HR:0.67, 95% CI 0.48 to 0.94).

### Association with cancer outcomes

BSO was associated with increased risk of second primary non-breast cancers in women having BSO≥55 years (HR:1.21, 95% CI 1.08 to 1.35), but there was no association in women who underwent BSO before age 55 years (HR 1.03, 95% CI 0.91 to 1.17). BSO at any age was not associated with CBC (HR 1.04, 95% CI 0.95 to 1.15).

### Association with mortality outcomes

Having BSO at or after the age of 55 years was associated with reduced risk of all-cause mortality with a HR of 0.92 (95% CI 0.87 to 0.96), but not in younger women (HR 1.03, 95% CI 0.98 to 1.08). BSO<55 years was associated with an increased risk of breast cancer mortality 1.09 (1.04–1.15), but no association was observed in women who had BSO after the age of 55 years (HR 0.96, 95% CI 0.89 to 1.05). Finally, BSO was associated with a reduction in the risk of non-breast cancer mortality for both women who had BSO<55 years (HR 0.86, 95% CI 0.78 to 0.96) and BSO≥55 years (HR 0.93, 95% CI 0.88 to 0.99).

### Sensitivity analyses

Sensitivity analysis for the associations in women who underwent the BSO≥55 years and restricted on women diagnosed with breast cancer at or after age of 55 years yielded similar results (supplementary materials 2: online supplemental table 6S).

Sensitivity analysis revealed that adjusting for hysterectomy primarily influenced the association between BSO performed before age of 55 years and breast cancer and all-cause mortalities. Among the ‘BSO<55 years’ analysis cohort hysterectomy alone and hysterectomy and BSO were associated with a reduction in the risks of all-cause mortality and breast cancer mortality. While BSO alone was associated with increased risk of breast cancer mortality (HR 1.26, 95% CI 1.19 to 1.34), all-cause mortality (HR 1.18, 95% CI 1.12 to 1.25) and reduced risk of non-breast cancer mortality (HR 0.82, 95% CI 0.71 to 0.96). More details in supplementary materials 2 (online supplemental figure 1S, table 4S, 5S)

When censoring at the date of CBC, BSO alone was not associated with breast cancer mortality with HR estimates of 1.05 (95% CI 0.97 to 1.14) and 0.90 (95% CI 0.73 to 1.11), for women who had BSO<55 years and≥55 years, respectively.

## Discussion

We used population-scale electronic health records to assess the association between BSO after breast cancer diagnosis and long-term health outcomes. This is the first time that HES-APC and the NCRD datasets were linked to answer this question in a cohort of patients with breast cancer diagnosed over a 24-year period.

BSO after breast cancer diagnosis was associated with an increased risk of CVD, IHD and depression in women who had BSO at any age and an increased risk of second non-breast cancer among women who had BSO at or after age 55 years. Investigating the CVD associations by the severity of the outcome showed no association with fatal CVD outcomes. The lack of significant association between BSO and fatal CVD outcomes, coupled with the observation that the increased risk of CVD and IHD seems to be primarily driven by an elevated risk of angina, suggests the possibility of detection bias influencing these associations.

BSO before the age of 55 years was associated with increased risk of breast cancer mortality, but reduced risk of non-breast cancer mortality. Although BSO after age of 55 years was not associated with breast cancer mortality, it was associated with a lower risk of death from other causes (non-breast cancer mortality) and lower all-cause mortality. A sensitivity analysis was conducted where CBC was considered a censoring event to assess the robustness of the finding that BSO in younger women was associated with increased breast cancer mortality. This analysis found no association between BSO in younger women and breast cancer mortality. It is therefore possible that the group of women who opted for BSO at a young age may be enriched for women who are genetically susceptible to breast cancer and who are at an increased risk for CBC.^19^ We explored this by stratifying the analysis on the indication for BSO (prophylactic/other benign indication), the indications were derived from the ICD-10 diagnosis codes recorded in HES at the date of having the BSO. The estimated HRs by indication were similar (supplementary materials 2; online supplemental table 7S).

BSO is indicated for various reasons including treatment of ovarian cancer, benign ovarian conditions affecting both ovaries, for example, endometriosis, ovarian cancer risk reduction in high-risk women (eg, *BRCA1* and *BRCA2* PV carriers)^20^ or for OFS in patients with breast cancer with ER-positive tumours.^6^ In this cohort, we censored women who had BSO for treatment of any gynaecological cancer or as a part of pelvic clearance procedure. Thus, the women in our cohort probably had the BSO for the other listed indications. It is also possible that the cohort included women who are *BRCA1* or *BRCA2* pathogenic variants (PVs) carriers given that the prevalence of both PVs among patients with breast cancer is around 2–3%.^21 22^

Several studies examined the association between OFS and all-cause mortality, disease-free survival (DFS) and other outcomes. OFS is recommended to be combined with tamoxifen or aromatase inhibitors in women with hormone-positive breast cancer. However, OFS could be achieved temporarily by GnRH agonist or permanently using radiation-induced ablation or BSO. Only BSO is recommended for ovarian cancer risk reduction. In a recent Cochrane systematic review and meta-analysis,^6^ medical OFS was associated with a significant reduction in the risk of all-cause mortality and DFS with HRs 0.80 (95% CI 0.71 to 0.89) and 0.81 (95% CI 0.75 to 0.88), respectively. While OFS by BSO was not associated with all-cause mortality (HR 0.86, 95% CI 0.57 to 1.28) or DFS (HR 0.96, 95% CI 0.70 to 1.30). It is important to note this systematic review answers a different question from our study which compares women who had BSO to women who might have had OFS through GnRH agonist or radiation-induced ablation.

Obermair *et al* examined the association between BSO after breast cancer diagnosis and mortality in two independent studies using the Queensland cancer registry (n=25 536)^8^ and the Western Australia cancer registry (n=15 395).^7^ In both studies, hysterectomy and BSO were associated with reduced risk of all-cause mortality and breast cancer mortality. However, BSO alone was not associated with all-cause mortality or breast cancer mortality. These findings are in line with our sensitivity analysis in which both hysterectomy alone and hysterectomy and BSO<55 years were associated with reduced risk of mortality, while BSO alone<55 years was associated with an increased risk of mortality.

The findings for the associations with CVD and all-cause mortality in the age-stratified analyses are in line with the associations in the general population. In a recent systematic review and meta-analysis on the long-term outcomes of BSO at the time of hysterectomy,^5^ hysterectomy and BSO were associated with increased risk of IHD in women who had the procedure before or after the age of 50 years and increased risk of all-cause mortality in women who had BSO before the age of 50 years. However, there was no association with all-cause mortality in women who had BSO after the age of 50 years.

These findings are important for counselling women with a personal history of breast cancer who are considering BSO. Guidance based on studies specifically conducted on this group would provide more informative support for decision-making than evidence derived from the general population.

The major strength of this study is the large sample size, up to date this is the largest study to examine the long-term outcomes of BSO after the diagnosis of breast cancer. The NCRD contains data on all patients with cancer diagnosed in England which minimises selection bias. We modelled the BSO and hysterectomy as time-dependent covariates to avoid immortal time bias. Also, we excluded women who had a history of any of the outcomes before the start of the follow-up.

Assessment of the association between BSO and long-term outcomes in electronic health records has several limitations. Unlike randomised controlled studies several factors (possibly confounders) influence the receipt or uptake of intervention in observational studies. To address this, we adjusted the analysis for a number of confounders including hormonal receptor status, treatment, age at diagnosis, deprivation index and Charlson Comorbidity Index. In addition, we censored women who had BSO for malignant indications to minimise confounding by indication of the BSO. However, there could be residual confounding resulting from the lack of information on confounders like family history of breast cancer, smoking status and body mass index. Also, detection bias could have influenced the results as women with a previous history of surgery might seek more medical attention. Nevertheless, we expect that this has not substantially biased the results as the cohort consists of patients with breast cancer who are likely to receive close medical monitoring. We attempted to minimise detection bias by starting the follow-up for BSO or hysterectomy 1 year following the surgery to avoid adding cases to the surgery groups who were accidentally discovered at the time of the surgery (prevalent cases). Another limitation is that non-cancer long-term outcomes were identified from hospital admissions in HES and from death certificates, which means we were only capable of identifying severe outcomes and this might have limited the power of the study to detect associations with certain outcomes such as dementia. This limitation may also explain the observed reduction in the risk of parkinsonism, which is not in line with previous findings.^23 24^ Additionally, the association with breast cancer mortality could be affected by the inaccurate coding of the cause of death. Finally, some tumour characteristic data were missing, for example, 29% of TNM stage and more than 50% of the ER status and HER2 status. To address this, we employed multiple imputation which has been shown to reduce bias in estimates compared with complete case analysis.^25^

## Conclusion

In women with a personal history of breast cancer, BSO before and after the age of 55 years is associated with an increased risk of long-term outcomes including CVD, cancer and depression. However, more work is needed to elucidate the possibility of confounding by family history and genetic susceptibility. Women with BSO above age of 55 years may benefit from a reduction in all-cause mortality.

## supplementary material

10.1136/bmjonc-2024-000574 online supplemental file 1

## Data Availability

Data may be obtained from a third party and are not publicly available.
