# Supplementary material for: Long-term outcomes of bilateral salpingo-oophorectomy in women with personal history of breast cancer
Source: BMJ Oncol. 2025 Feb 25;4(1):e000574. doi: 10.1136/bmjonc-2024-000574 (PMC11880784; doi:10.1136/bmjonc-2024-000574)
Supplement: online supplemental file 1 [file bmjonc-4-1-s001.docx]

# Table 1S: ICD10 codes identified from HES or DEATHCAUSE_1A for each of the long-term outcomes

| Outcome | ICD10 code |
| --- | --- |
| Total cardiovascular diseases | I10-I15, I20-I25, I26-I28,  I30-I52, I60-I69, I70-I79,  F01, G45, R96 |
| Ischaemic heart disease | I20-I25 |
| Total cerebrovascular diseases | I60-I69 |
| Stroke | I60-I64 |
| Ischaemic stroke | I63 |
| Haemorrhagic stroke | I60-I62 |
| Dementia | A81.0, F00-F03, F05.1, F10.6, G30, G31.0, G31.1, G31.8, I67.3 |
| Depression | F32, F33, F34.0, F34.1, F41, X67-69, X7, X80-84, Y870 |
| Parkinsonism | G20-G26 |

# Multiple imputation of the missing data

We used multivariate imputations by chained equations to impute the missing TNM stage, grade, ER status, HER2 status, tumour size, number of lymph nodes excised, ethnicity and Charlson-comorbidity index. The methods used to predict the missing observations were multinomial logistic regression for TNM stage, grade and ethnicity, binary logistic regression for ER status and HER2 status and predictive mean matching for tumour size and number of lymph nodes excised. The variables included in each imputation model were the age at breast cancer diagnosis, year of diagnosis, ethnicity, death event indicator, Nelson-Aalen estimator, which is a non-parametric estimator of cumulative hazard rate and recommended to be included in imputation models used for survival analysis[1], TNM stage, grade, tumour size, number of excised lymph nodes, ER status, HER2 status and treatment flags for the receipt of chemotherapy, radiotherapy or immunotherapy within the first year after the breast cancer diagnosis. Each imputation model generated 12 complete datasets through 7 iterations. We examined the convergence plots to verify convergence of the imputation process (Figure 1S).

Figure 1S: Convergence plots


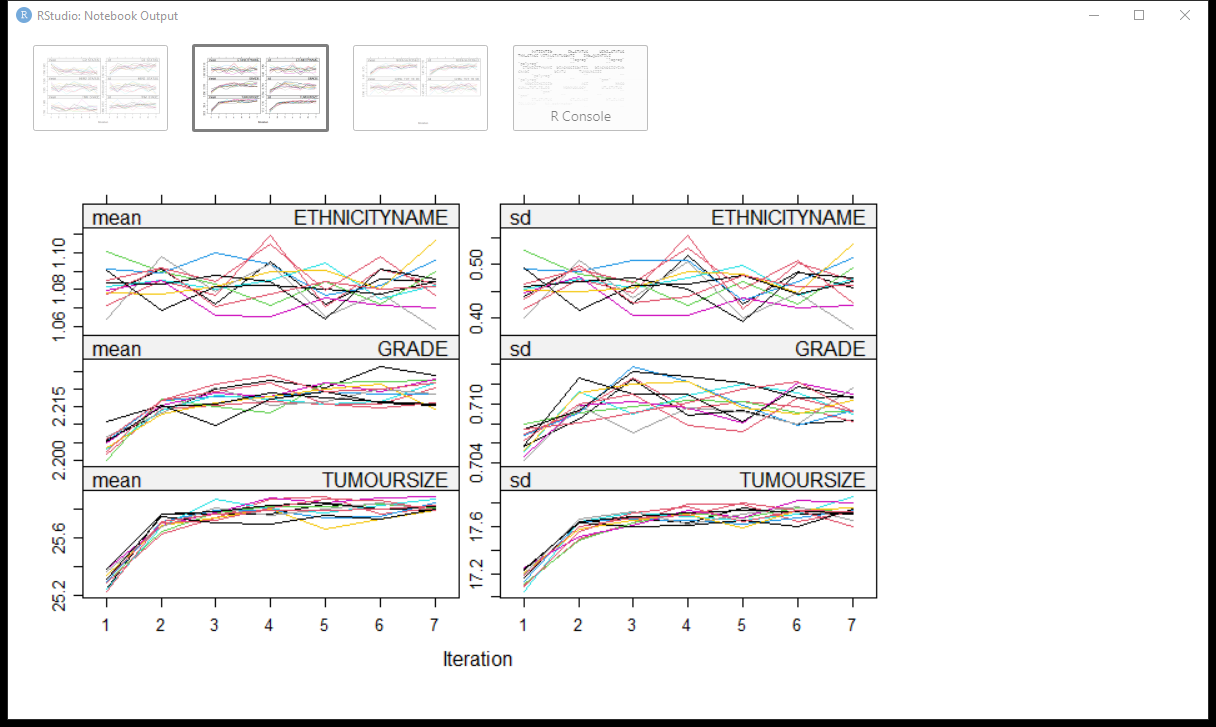

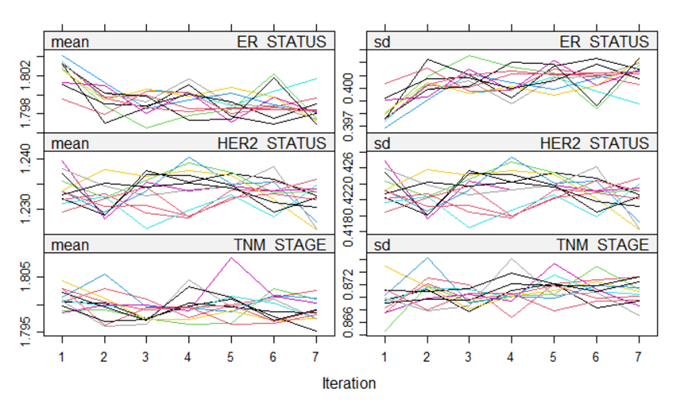

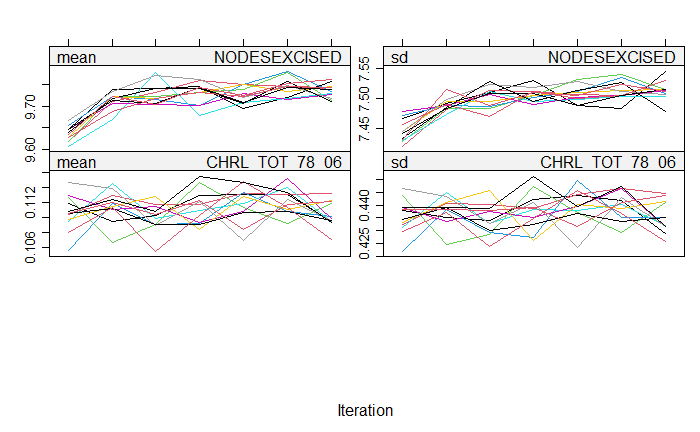


Table 2S: Associations between BSO and long-term outcomes by age at BSO adjusted for hysterectomy

| Outcome | | Age at BSO | N at risk | N events | Person years | HR (95% CI) |
| --- | --- | --- | --- | --- | --- | --- |
| All-cause mortality | | Unstratified | 547,310 | 158,447 | 5,108,170 | 1.16 (1.11-1.21) |
|  |  | <55 | 231,306 | 50,053 | 2,304,019 | 1.17 (1.11-1.23) |
|  |  | ≥55 | 532,181 | 156,083 | 4,947,996 | 1.01 (0.95-1.07) |
| Total CVD | Total | Unstratified | 415,485 | 104,491 | 3,570,242 | 1.10 (1.05-1.16) |
|  | Non-fatal |  |  | 100,279 | 3,570,245 | 1.10 (1.05-1.16) |
|  | Fatal |  |  | 11,546 | 4,157,063 | 1.06 (0.90-1.25) |
|  | Total | <55 | 206,942 | 33,189 | 1,935,037 | 1.07 (1.00-1.14) |
|  | Non-fatal |  |  | 32,070 | 1,935,038 | 1.08 (1.01-1.15) |
|  | Fatal |  |  | 2,153 | 2,119,670 | 0.91 (0.69-1.21) |
|  | Total | ≥55 | 401,756 | 102,312 | 3,434,178 | 1.09 (1.01-1.16) |
|  | Non-fatal |  |  | 98,144 | 3,434,181 | 1.09 (1.02-1.17) |
|  | Fatal |  |  | 11,461 | 4,008,443 | 1.00(0.83-1.22) |
| IHD | Total | Unstratified | 525,665 | 28,178 | 4,805,042 | 1.09 (0.99-1.21) |
|  | Non-fatal |  |  | 25,710 | 4,805,043 | 1.11 (1.00-1.23) |
|  | Fatal |  |  | 3,549 | 4,951,005 | 0.96 (0.69-1.35) |
|  | Total | <55 | 229,018 | 5,871 | 2,250,590 | 1.06 (0.90-1.25) |
|  | Non-fatal |  |  | 5,556 | 2,250,590 | 1.06 (0.90-1.25) |
|  | Fatal |  |  | 428 | 2,285,240 | 0.90 (0.45-1.78) |
|  | Total | ≥55 | 510,653 | 27,873 | 4,647,631 | 1.14 (1.01-1.29) |
|  | Non-fatal |  |  | 25,418 | 4,647,632 | 1.17 (1.03-1.33) |
|  | Fatal |  |  | 3,534 | 4,791,818 | 0.96 (0.66-1.41) |
| Cerebrovascular diseases | Total | Unstratified | 540,517 | 20,141 | 5,002,065 | 1.08 (0.96-1.22) |
|  | Non-fatal |  |  | 18,203 | 5,002,068 | 1.08 (0.95-1.23) |
|  | Fatal |  |  | 4,106 | 5,068,176 | 1.03 (0.76-1.39) |
|  | Total | <55 | 230,308 | 3,512 | 2,282,799 | 0.88 (0.71-1.09) |
|  | Non-fatal |  |  | 3,207 | 2,282,800 | 0.86 (0.69-1.08) |
|  | Fatal |  |  | 518 | 2,296,864 | 1.03 (0.60-1.77) |
|  | Total | ≥55 | 525,428 | 19,980 | 4,842,903 | 1.08 (0.93-1.25) |
|  | Non-fatal |  |  | 18,052 | 4,842,906 | 1.09 (0.94-1.27) |
|  | Fatal |  |  | 4,085 | 4,908,357 | 0.89 (0.63-1.25) |
| Haemorrhagic stroke | | Unstratified | 543,914 | 4,584 | 5,076,992 | 1.05 (0.83-1.35) |
|  |  | <55 | 230,728 | 1,101 | 2,296,233 | 0.97 (0.68-1.39) |
|  |  | ≥55 | 528,808 | 4,530 | 4,917,201 | 0.97 (0.70-1.34) |
| Ischaemic stroke | | Unstratified | 543,914 | 9,971 | 5,055,430 | 1.12 (0.94-1.33) |
|  |  | <55 | 230,728 | 1,513 | 2,294,263 | 0.81 (0.58-1.13) |
|  |  | ≥55 | 528,808 | 9,902 | 4,895,713 | 1.18 (0.97-1.44) |
| Parkinsonism | | Unstratified | 545,231 | 3,533 | 5,082,902 | 0.73 (0.53-0.98) |
|  |  | <55 | 230,926 | 648 | 2,298,880 | 0.86 (0.54-1.40) |
|  |  | ≥55 | 530,135 | 3,493 | 4,923,142 | 0.62 (0.42-0.92) |
| Dementia | | Unstratified | 545,933 | 12,036 | 5,072,461 | 0.87 (0.73-1.04) |
|  |  | <55 | 231,212 | 625 | 2,301,234 | 0.94 (0.52-1.70) |
|  |  | ≥55 | 530,804 | 12,016 | 4,912,385 | 0.89 (0.74-1.06) |
| Depression | | Unstratified | 506,423 | 21,243 | 4,784,915 | 1.13 (1.04-1.23) |
|  |  | <55 | 213,588 | 10,491 | 2,150,730 | 1.09 (0.99-1.21) |
|  |  | ≥55 | 492,741 | 20,103 | 4,640,259 | 1.12 (0.97-1.29) |
| Contralateral breast cancer | | Unstratified | 522802 | 13948 | 4,071,952 | 1.08 (0.96-1.23) |
|  |  | <55 | 221647 | 6819 | 1,799,643 | 1.08 (0.93-1.26) |
|  |  | ≥55 | 508460 | 13179 | 3,967,272 | 1.14 (0.93-1.38) |
| Breast cancer mortality | | Unstratified | 547,246 | 80,795 | 5,107,400 | 1.24 (1.18-1.31) |
|  |  | <55 | 231,281 | 36,745 | 2,303,692 | 1.25 (1.18-1.32) |
|  |  | ≥55 | 532,118 | 78,865 | 4,947,241 | 1.03 (0.93-1.14) |

Table 3S: Association between BSO (age at surgery <55 and ≥55) and breast cancer mortality with and without censoring for contralateral breast cancer

| Age at BSO | Model 1: HR (95% CI) | Model 2: HR (95% CI) |
| --- | --- | --- |
| <55 | 1.10 (1.04-1.16) | 0.90 (0.84-0.96) |
| ≥55 | 0.99 (0.91-1.09) | 1.02 (0.91-1.13) |

*Model 1: association between BSO and breast cancer mortality with study’s specified censoring*

*Model 2: association between BSO and breast cancer mortality with censoring for contralateral breast cancer.*

*Hazard ratios adjusted for age at breast cancer diagnosis, year of diagnosis, tumour size, number of excised lymph nodes, M-stage, grade, ER status, HER2 status, ethnicity, deprivation index and Charlson comorbidity index*

## Adjustment for hysterectomy

Figure 1S shows the log (HR) for the associations between BSO and long-term outcomes before and after the adjustment for hysterectomy. All hazard ratios adjusted for hysterectomy are shown in Table1S. Adjustment for hysterectomy yielded significant association between BSO and 2^nd^ non breast cancer with HR of 1.21 (95%CI: 1.08-1.35). In addition, there was an increased risk of all-cause mortality, breast cancer mortality and non-breast cancer mortality.  Table 4 shows the association between surgery types (hysterectomy, BSO and hysterectomy and BSO) and all-cause mortality, breast cancer mortality, non-breast cancer mortality and 2^nd^ non-breast cancer.

For the BSO <55 analysis cohort hysterectomy and hysterectomy and BSO were associated with reduction in the risk of all-cause mortality, but BSO was associated with increased risk of all-cause mortality (HR:1.18, 95%CI:1.12-1.25). The same association pattern was observed with breast cancer mortality and the association with BSO yielded a hazard ratio of 1.26 (95%CI:1.19-1.34). Hysterectomy alone and BSO alone were associated with reduction in the risk of non-breast cancer mortality with hazard ratios of 0.75 (95%CI:0.61-0.91) and 0.82 (0.71-0.96), while hysterectomy and BSO showed reduction in the risk of non-breast cancer mortality, but was not statistically significant (HR:0.89, 95%CI:0.78-1.02). Furthermore, hysterectomy and hysterectomy and BSO were associated with reduction and the risk of 2nd non-breast cancer with hazard ratios of 0.72 (95%CI:0.58-0.89) and 0.86 (95%CI:0.74-0.99), while there was no association between BSO and 2nd non breast cancer (HR:0.98, 95%CI:0.84-1.13).

Whereas in the BSO ≥ 55 analysis cohort hysterectomy alone (HR:0.80, 95%CI:0.75-0.85) and BSO alone (HR:0.79, 95%CI:0.72-0.88) were associated with reduced risk of all-cause mortality, while hysterectomy coupled with BSO didn’t yield the same significant reduction in the risk of all-cause mortality (HR:0.96, 95%CI:0.90-1.02). Similar association pattern was observed with the association with breast cancer mortality, with hazard ratio of 0.80 (95%CI:0.68-0.93) for the association with BSO. For non-breast cancer mortality hysterectomy showed significant reduction in the risk with HR 0.79 (95%CI:0.74-0.85), no significant association was detected with BSO or hysterectomy and BSO. Finally, hysterectomy was associated with reduced risk of 2^nd^ non-breast cancers (HR: 0.74, 95%CI:0.66-0.83), while BSO and BSO and hysterectomy were not significantly associated with increased risk of 2nd non-breast cancer.

Figure 2S: Association between BSO by age at surgery and the long-term outcomes both unadjusted and unadjusted for hysterectomy.


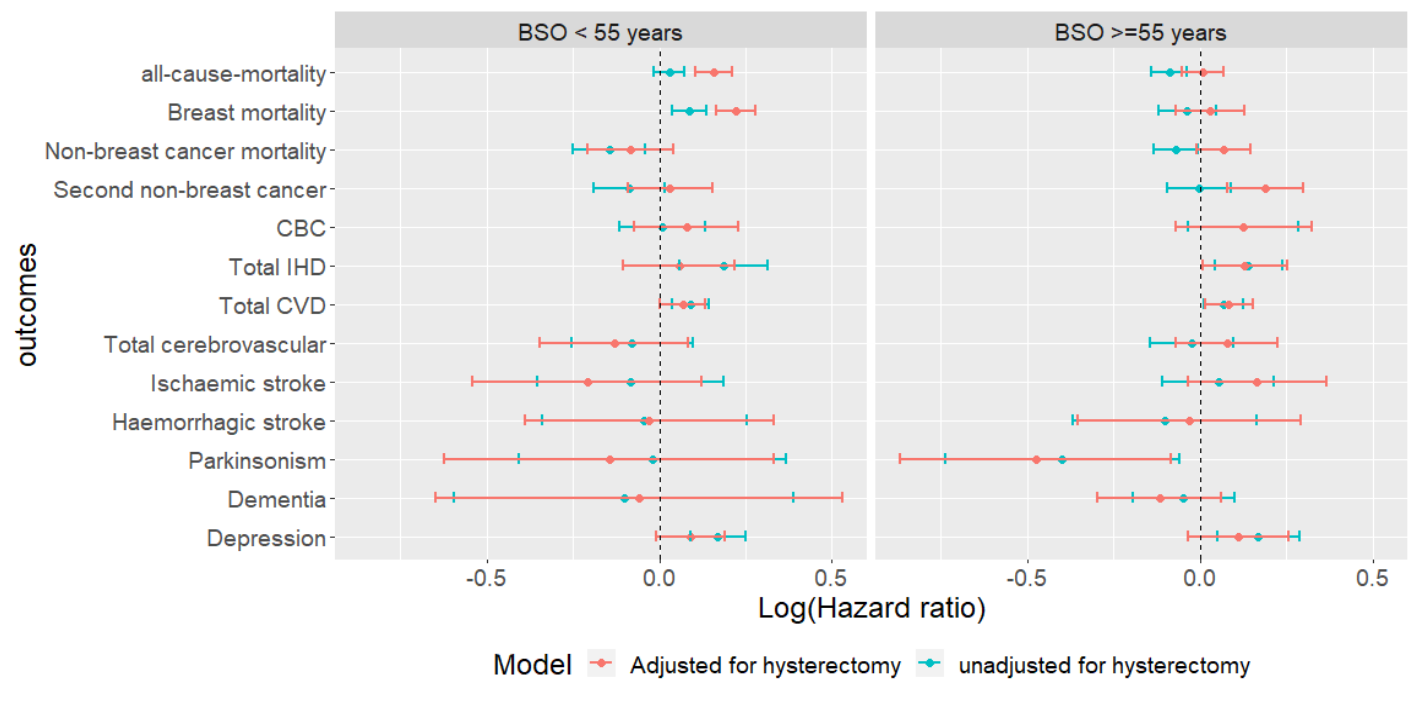


*Points are for Log (HR) and error bars are for the confidence intervals for the association between BSO and the long-term outcomes. Blue is for models unadjusted for hysterectomy and red is for the models adjusted for hysterectomy. Hazard ratios were additionally adjusted for age at breast cancer diagnosis, year of diagnosis, tumour size, number of excised lymph nodes, M-stage, grade, ER status, HER2 status, ethnicity, deprivation index and Charlson comorbidity index.*

Table 4S: Association between surgery (hysterectomy, hysterectomy and BSO, BSO) and all-cause mortality, breast cancer mortality, non-breast cancer mortality and 2^nd^ non-breast cancer

| Surgery type | Age at surgery | All-cause mortality | Breast cancer mortality | Non-breast cancer mortality | 2^nd^ non-breast cancer | |
| --- | --- | --- | --- | --- | --- | --- |
|  |  | **Hazard ratio (95% CI)** | | | | |
| None  Hysterectomy  BSO  Hysterectomy & BSO | <55 | 1 (Reference)  0.75 (0.66-0.86)  1.18 (1.12-1.25)  0.84 (0.77-0.90) | 1 (Reference)  0.75 (0.63-0.89)  1.26 (1.19-1.34)  0.84 (0.77-0.92) | 1 (Reference)  0.75 (0.61-0.91)  0.82 (0.71-0.96)  0.89 (0.78-1.02) | | 1 (Reference)  0.72 (0.58-0.89)  0.98 (0.84-1.13)  0.86 (0.74-0.99) |
| None  Hysterectomy  BSO  Hysterectomy & BSO | ≥55 | 1 (Reference)  0.80 (0.75-0.85)  0.79 (0.72-0.88)  0.96 (0.90-1.02) | 1 (Reference)  0.80 (0.73-0.89)  0.80 (0.68-0.93)  1.04 (0.94-1.15) | 1 (Reference)  0.79 (0.74-0.85)  0.90 (0.79-1.03)  0.94 (0.87-1.01) | | 1 (Reference)  0.74 (0.66-0.83)  1.12 (0.94-1.32)  0.95 (0.85-1.06) |

The sensitivity analysis for the association between surgery type and breast cancer mortality with censoring at the date of CBC is shown in Table 5S. In the analysis censoring at the date of CBC showed that BSO alone is not associated with increased risk of breast cancer mortality (HR:1.05, 95%CI:0.97-1.14).

Table 5S: Association between surgery type (hysterectomy, BSO, hysterectomy and BSO) and breast cancer mortality with and without censoring for contralateral breast cancer

| Surgery type | Age at surgery | CBC* | Breast cancer mortality | |
| --- | --- | --- | --- | --- |
|  |  | HR (95% CI) | Model 1: HR (95%CI) | Model 2: HR (95%CI) |
| None  Hysterectomy  BSO  Hysterectomy & BSO | <55 | 1 (Reference)  0.99 (0.76-1.28)  1.14 (0.96-1.35)  0.89 (0.75-1.07) | 1 (Reference)  0.77 (0.64-0.92)  1.29 (1.21-1.38)  0.83 (0.76-0.92) | 1 (Reference)  0.71 (0.58-0.86)  1.05 (0.97-1.14)  0.60 (0.61-0.78) |
| None  Hysterectomy  BSO  Hysterectomy & BSO | ≥55 | 1 (Reference)  1.03 (0.87-1.23)  1.31 (0.97-1.78)  1.08 (0.89-1.30) | 1 (Reference)  0.81 (0.73-0.91)  0.83 (0.70-1.00)  1.07 (0.96-1.19) | 1 (Reference)  0.82 (0.72-0.92)  0.90 (0.73-1.11)  1.06 (0.94-1.19) |

**CBC: Contralateral breast cancer.*

*Model 1: association between BSO and breast cancer mortality with study’s specified censoring*

*Model 2: association between BSO and breast cancer mortality with censoring for contralateral breast cancer.*

*Hazard ratios adjusted for age at breast cancer diagnosis, year of diagnosis, tumour size, number of excised lymph nodes, M-stage, grade, ER status, HER2 status, ethnicity, deprivation index and Charlson comorbidity index*

## Table 6S: Sensitivity analysis for BSO ≥55

| Outcome | HR (95%CI) |
| --- | --- |
| All-cause mortality | 0.96 (0.90-1.01) |
| Total CVD | 1.03 (0.96-1.10) |
| IHD | 1.11 (1.00-1.25) |
| Cerebrovascular disease | 0.97 (0.85-1.11) |
| 2^nd^ non-breast cancer | 1.13 (1.01-1.26) |
| Contralateral breast cancer | 1.10 (0.90-1.35) |
| Breast cancer mortality | 0.99 (0.89-1.10) |
| Dementia | 0.94 (0.80-1.09) |
| Depression | 1.11 (0.95-1.30) |
| Parkinsonism | 0.62 (0.42-0.92) |

# Schoenfeld residuals to assess the proportional hazard assumption of the Cox-regression models

Figure 3S: All-cause mortality, BSO ≥55

*
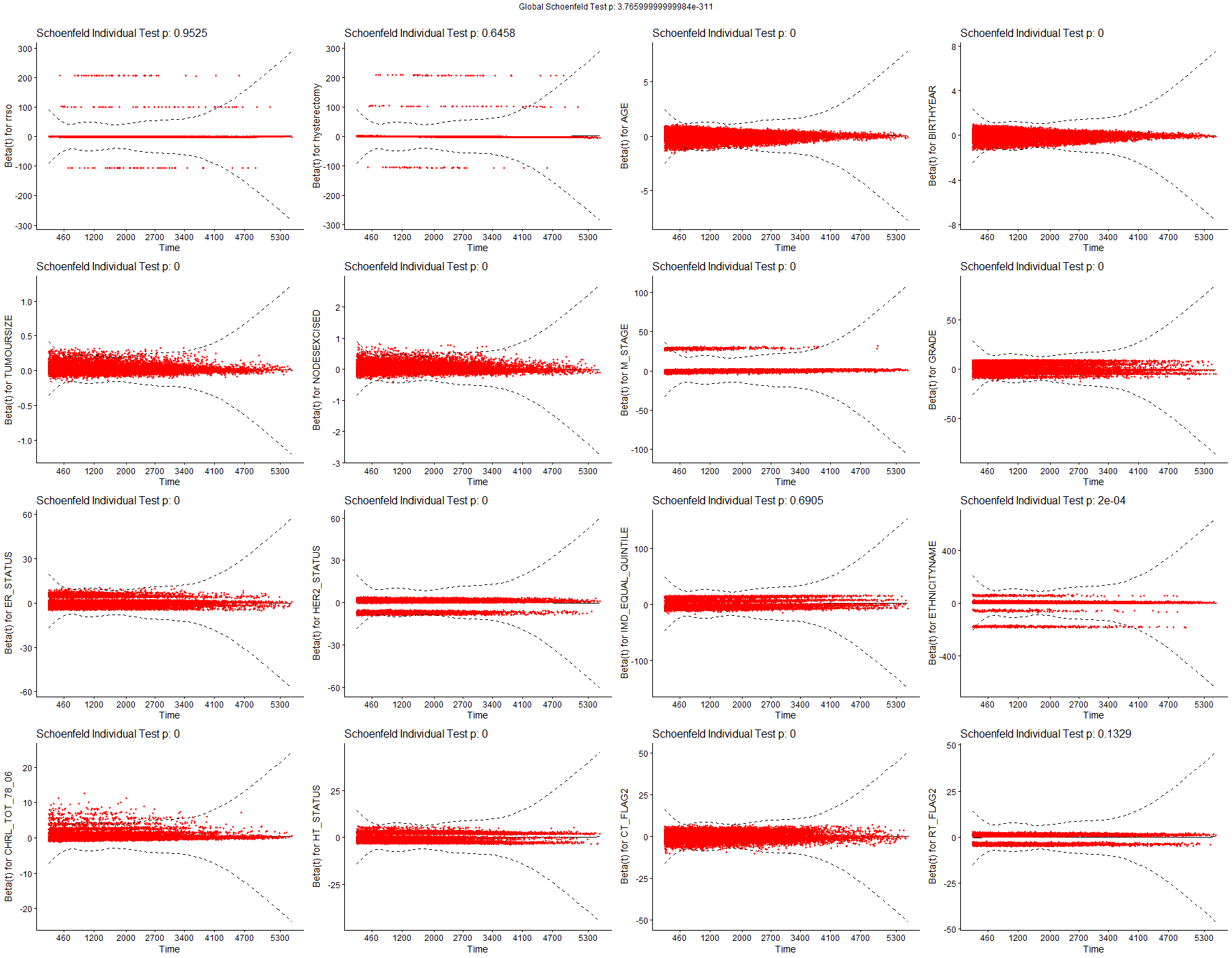
*

Figure 4S: all-cause mortality, BSO <55

*
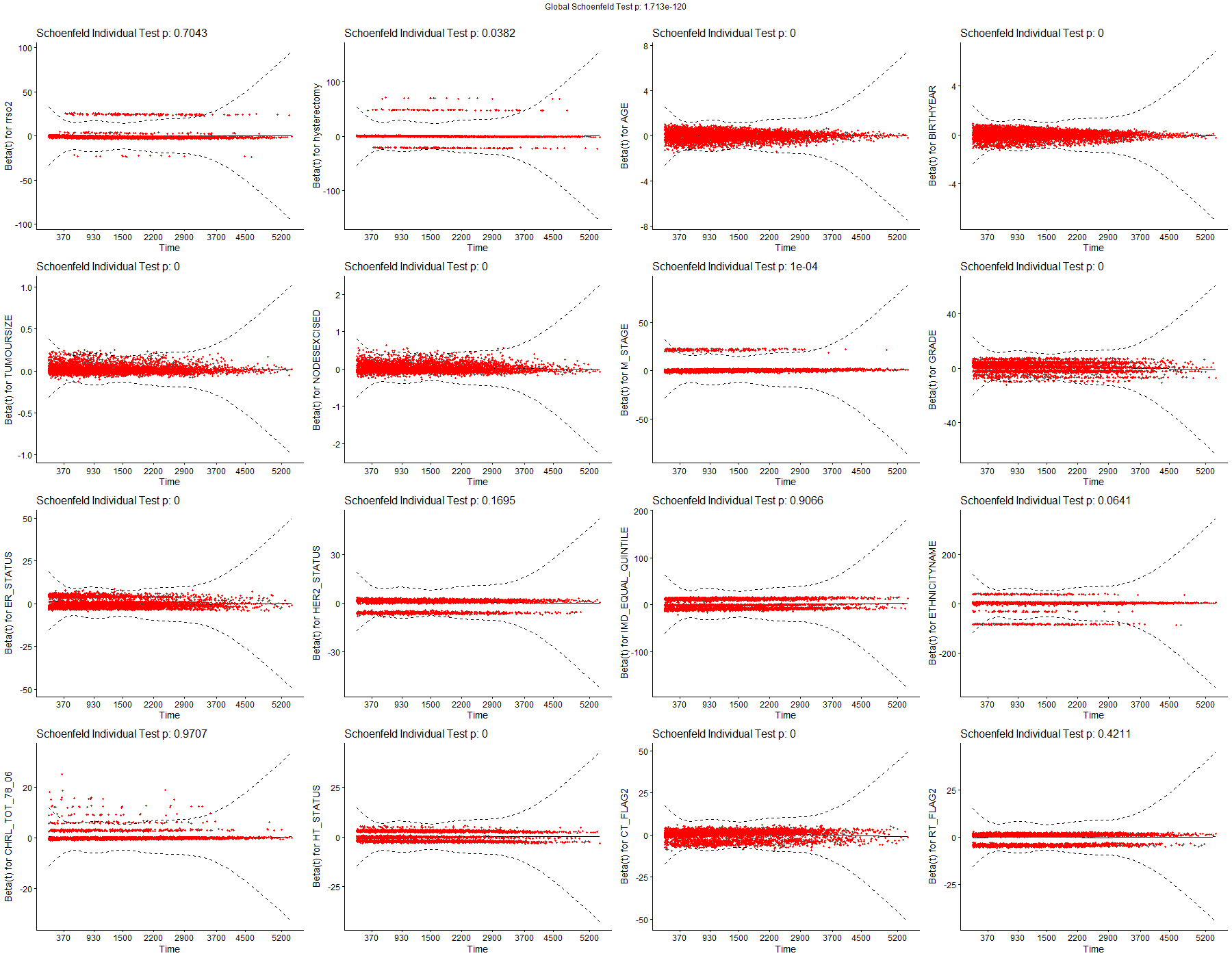
*

Figure 5S: Total CVD, BSO ≥55


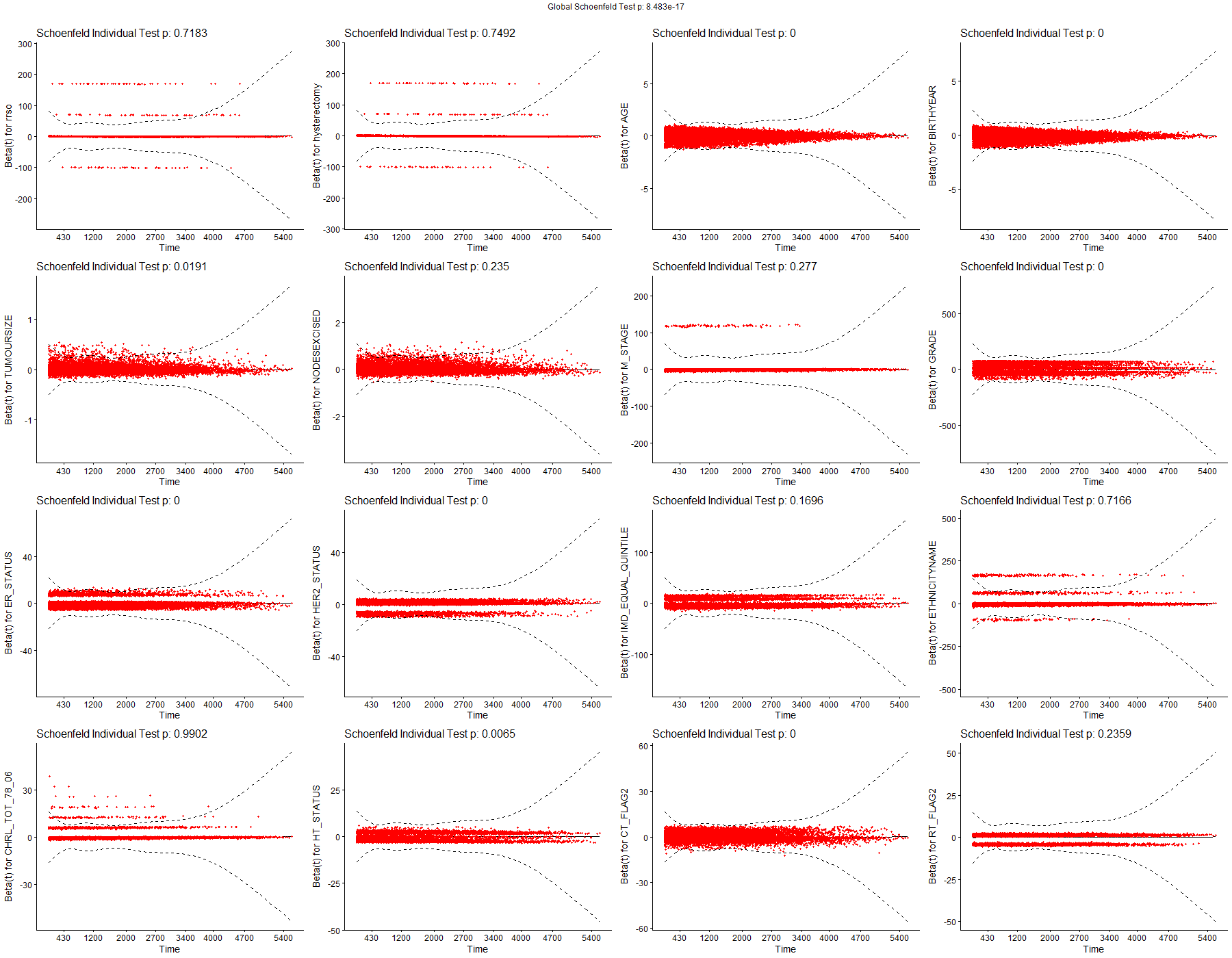


Figure 6S: Total CVD, BSO<55


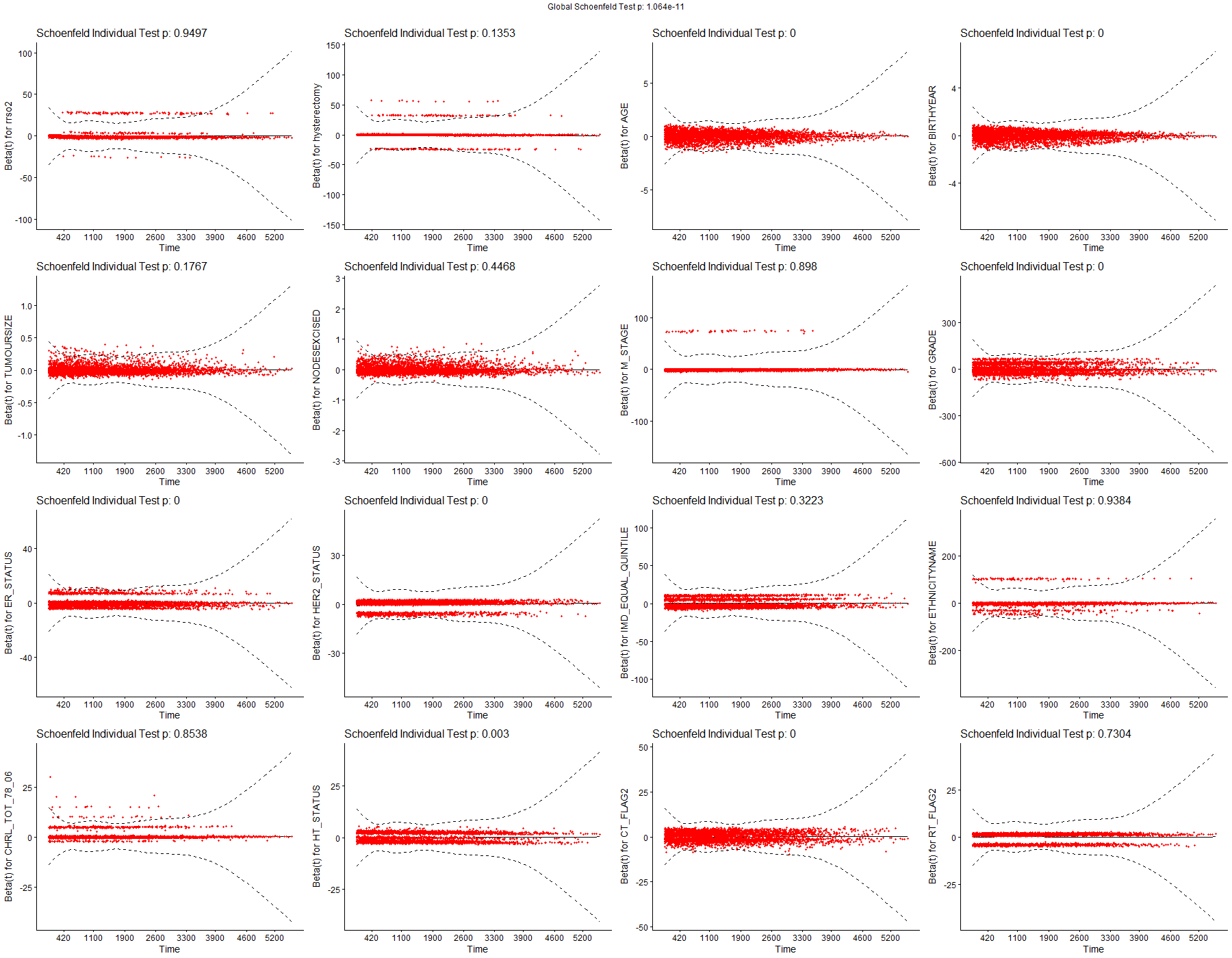


Figure 7S: 2nd non-breast cancer, BSO≥55


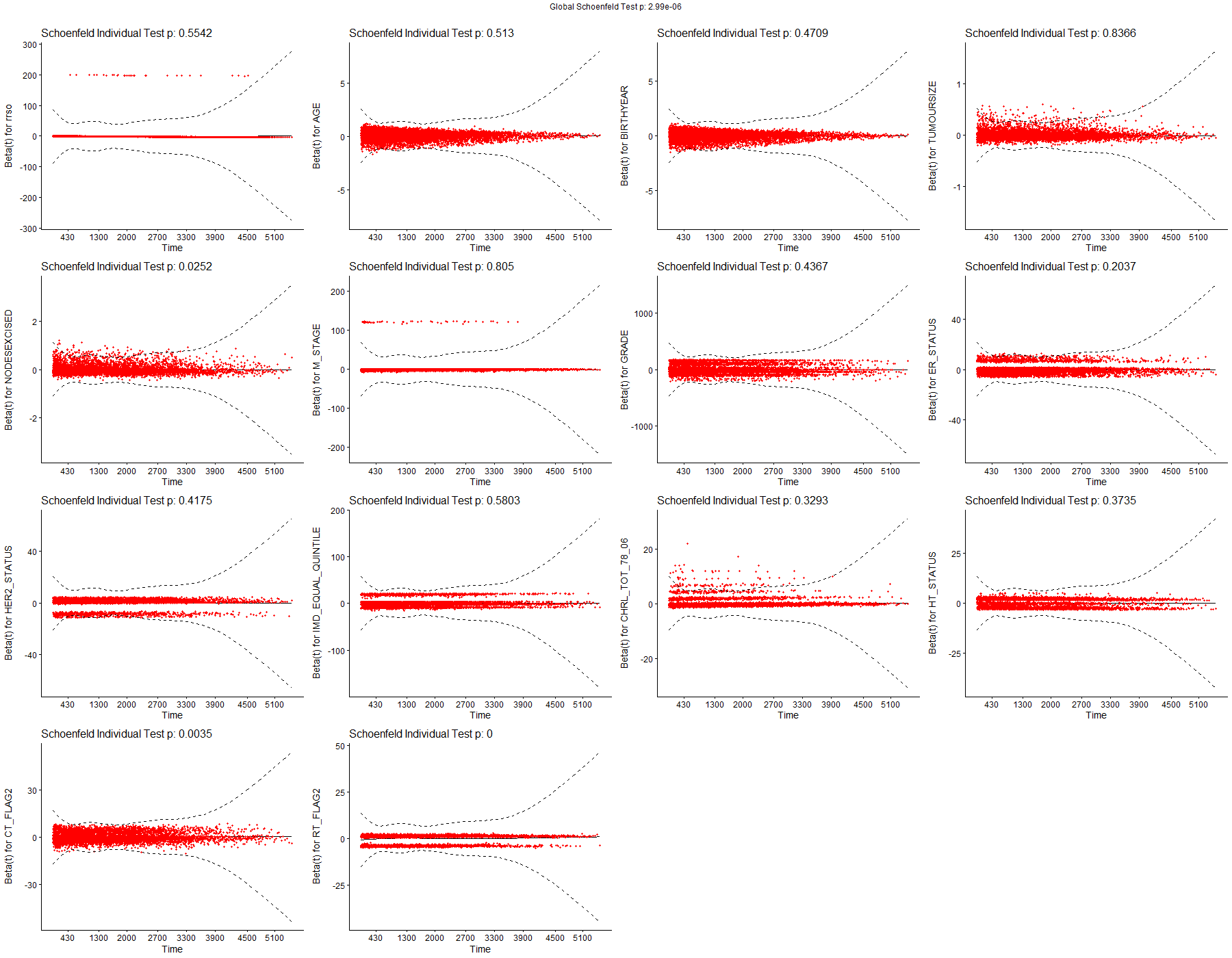


Figure 8S: 2nd non-breast cancer, BSO<55


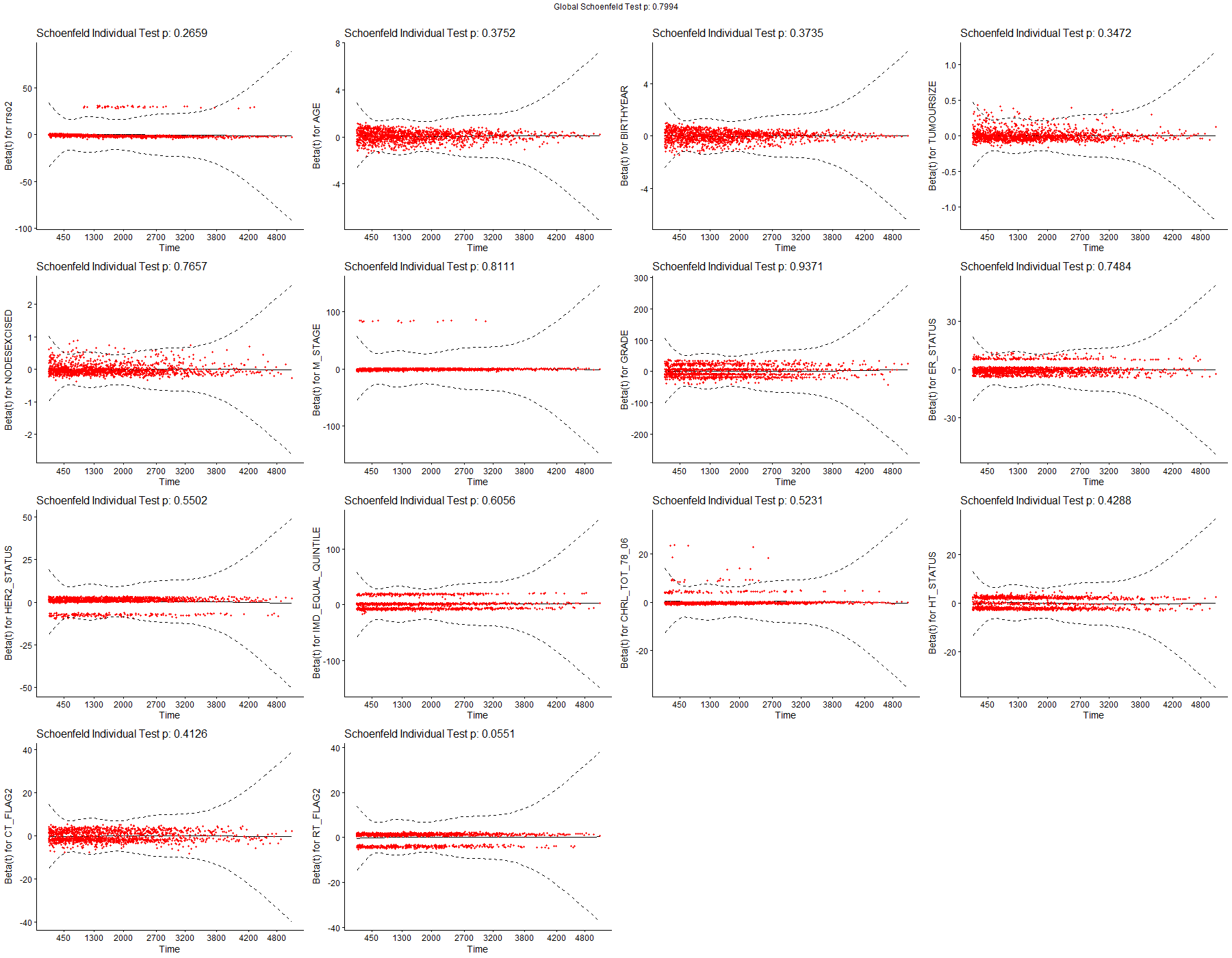


Figure 9S: Breast cancer mortality, BSO≥55


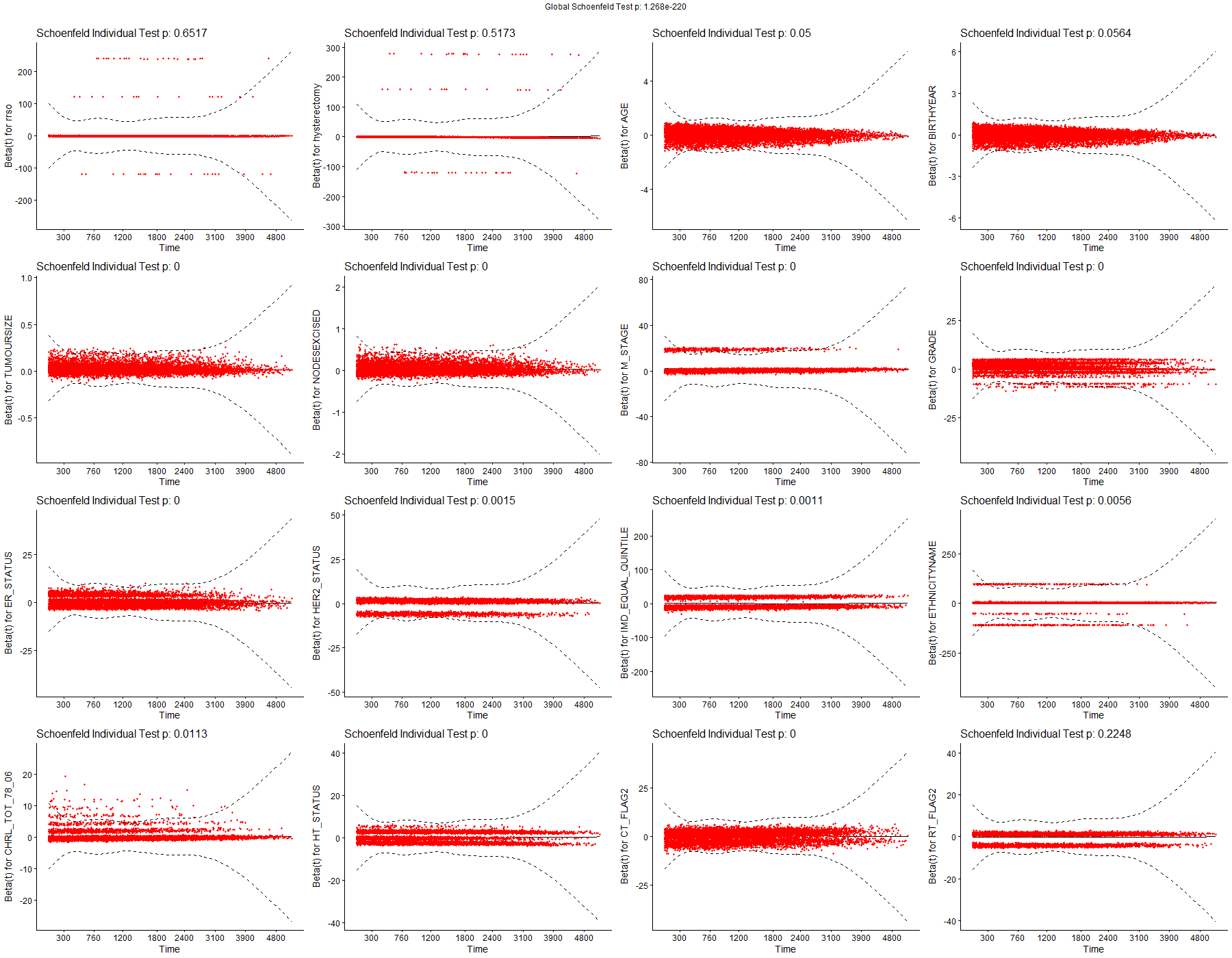


Figure 10S: Breast cancer mortality, BSO<55


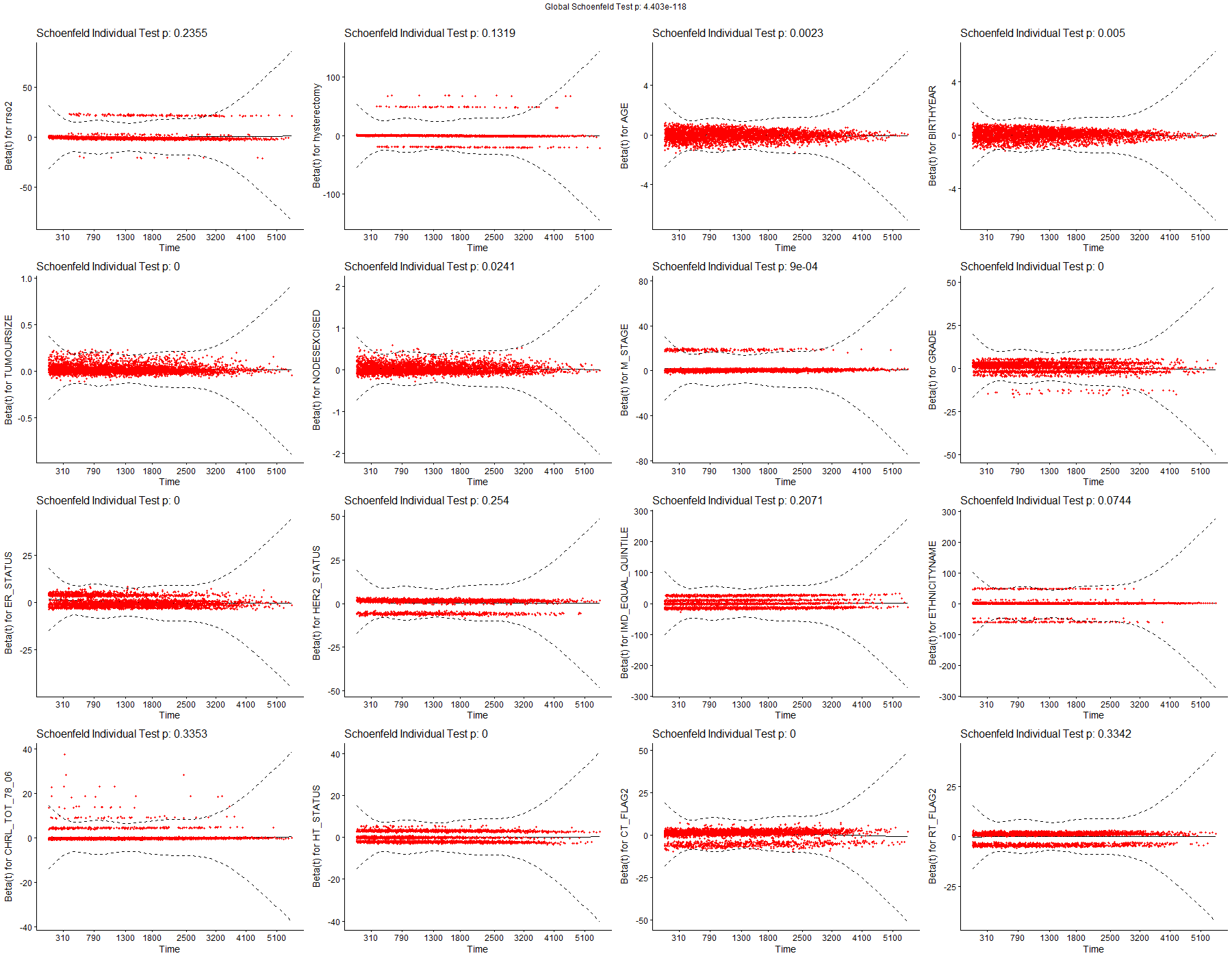


Figure 11S: Contralateral breast cancer analysis, BSO≥55


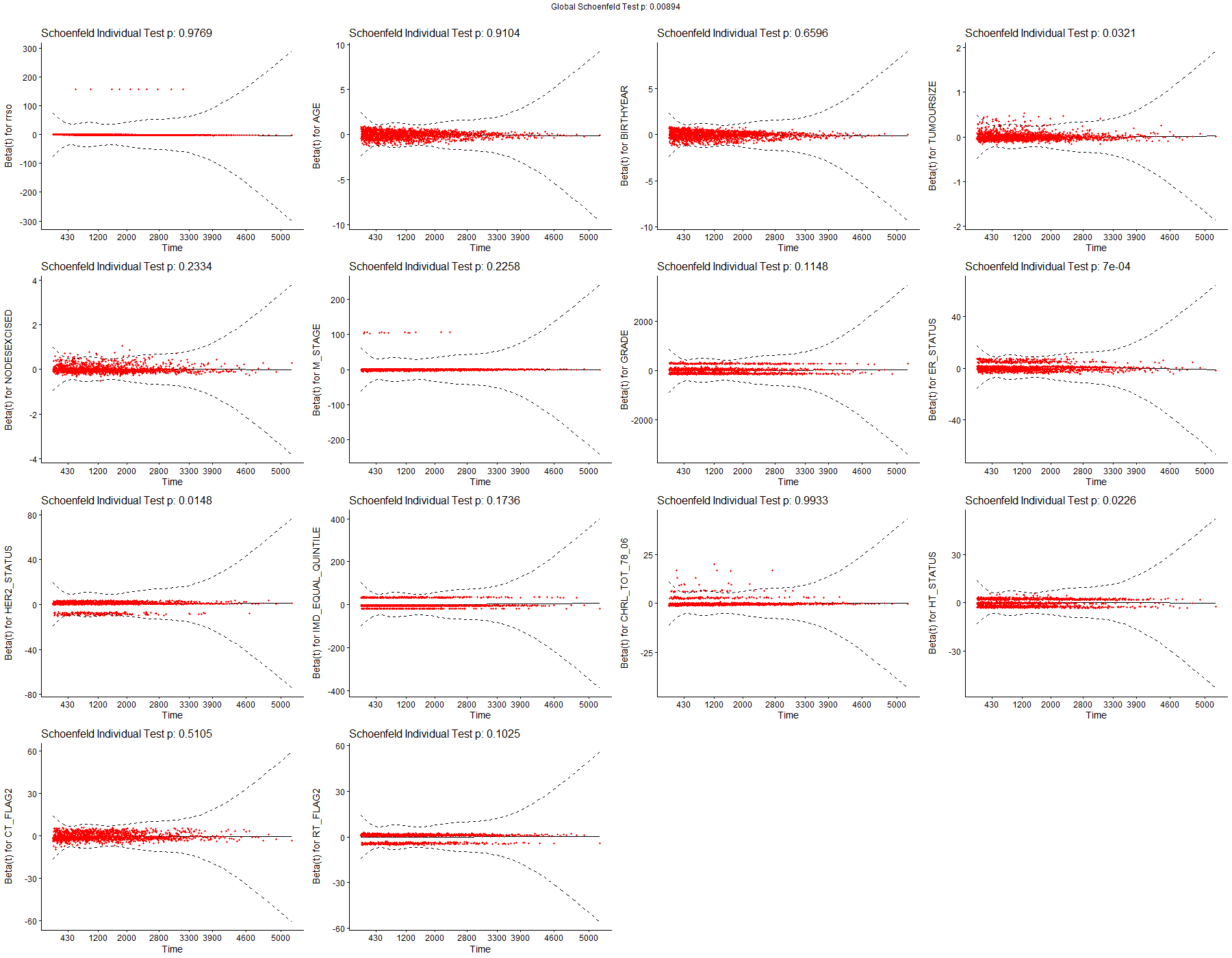


Figure 12S: Contralateral breast cancer, BSO<55


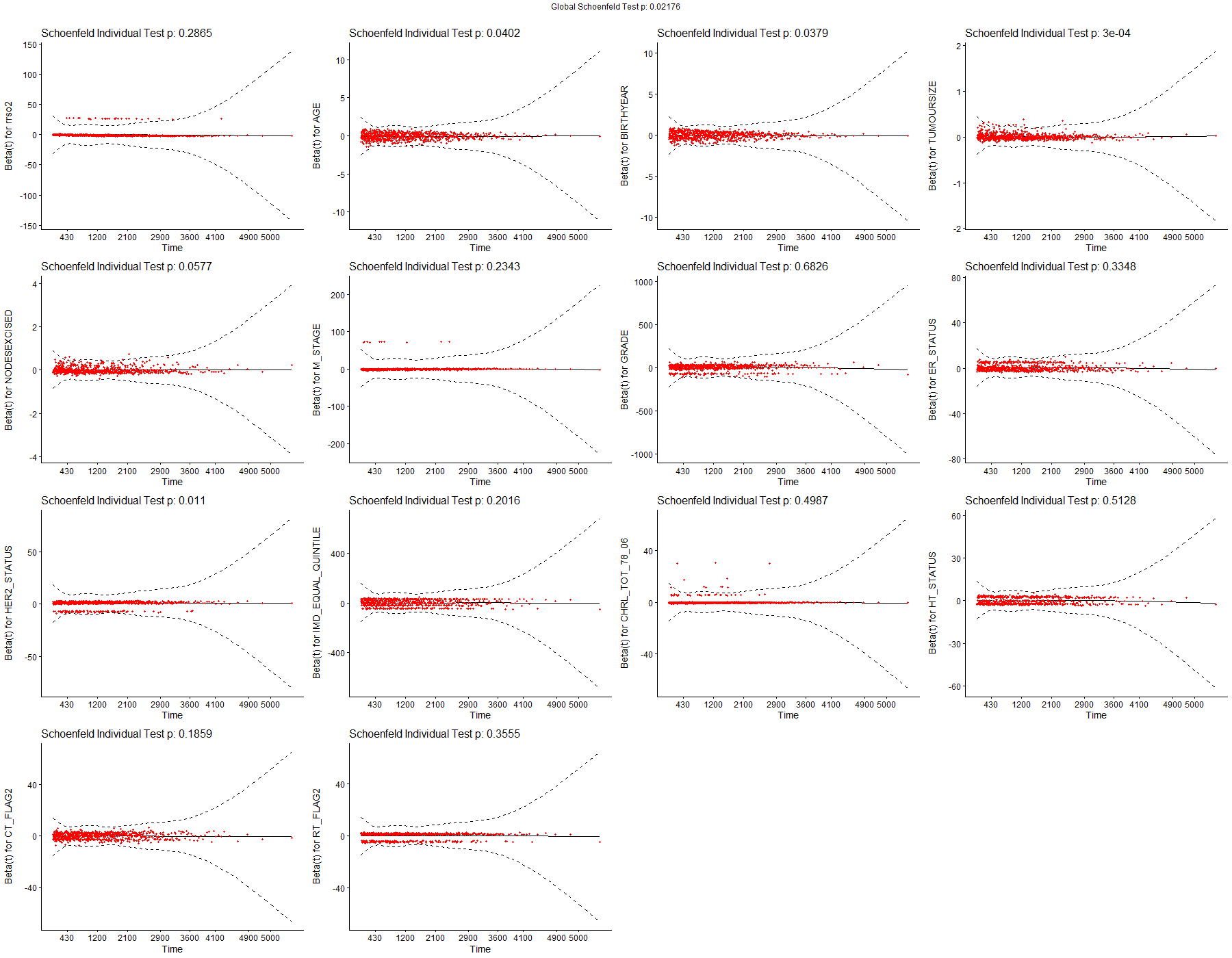


Figure 13S: Dementia, BSO≥55


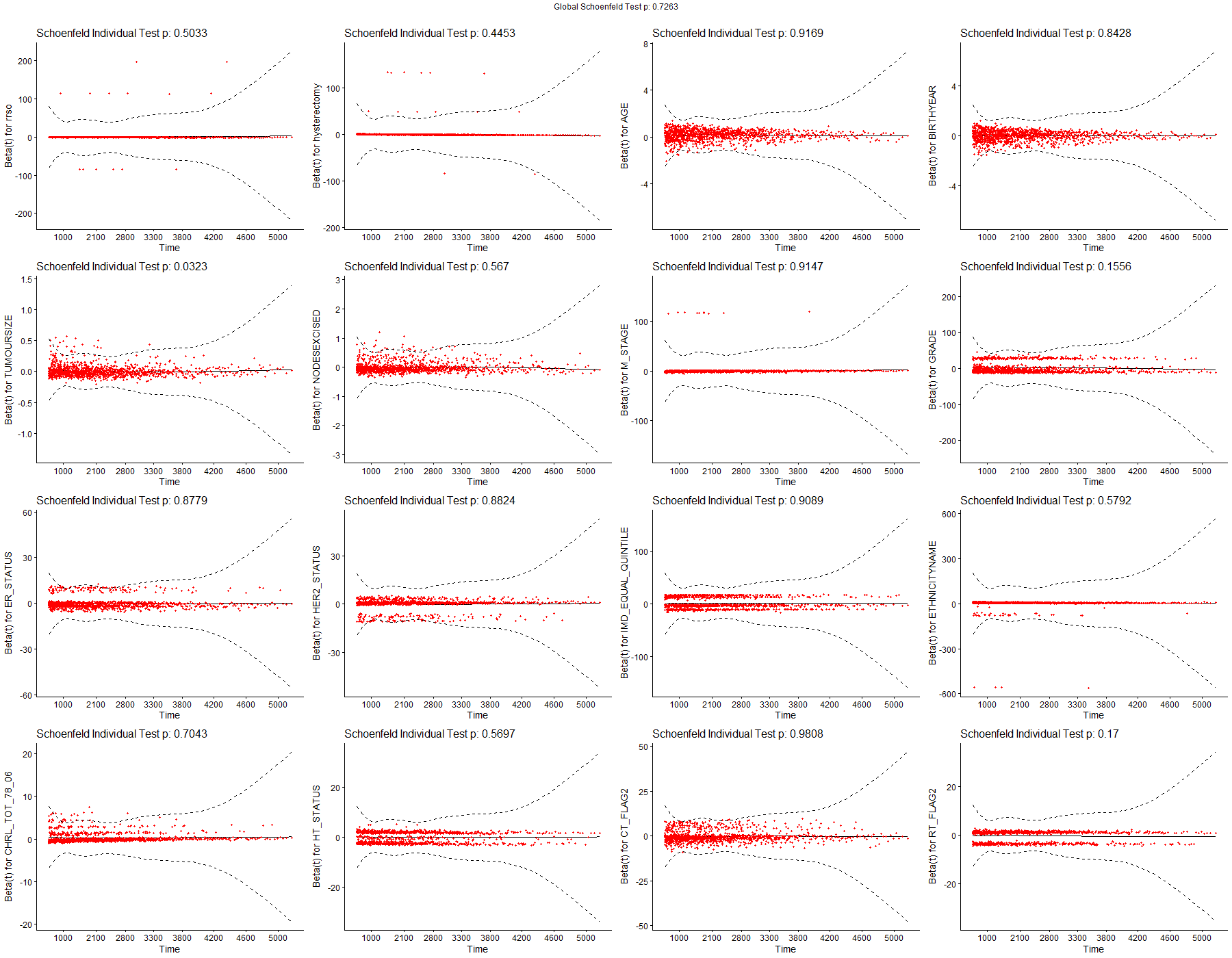


Figure 14S: Dementia, BSO<55


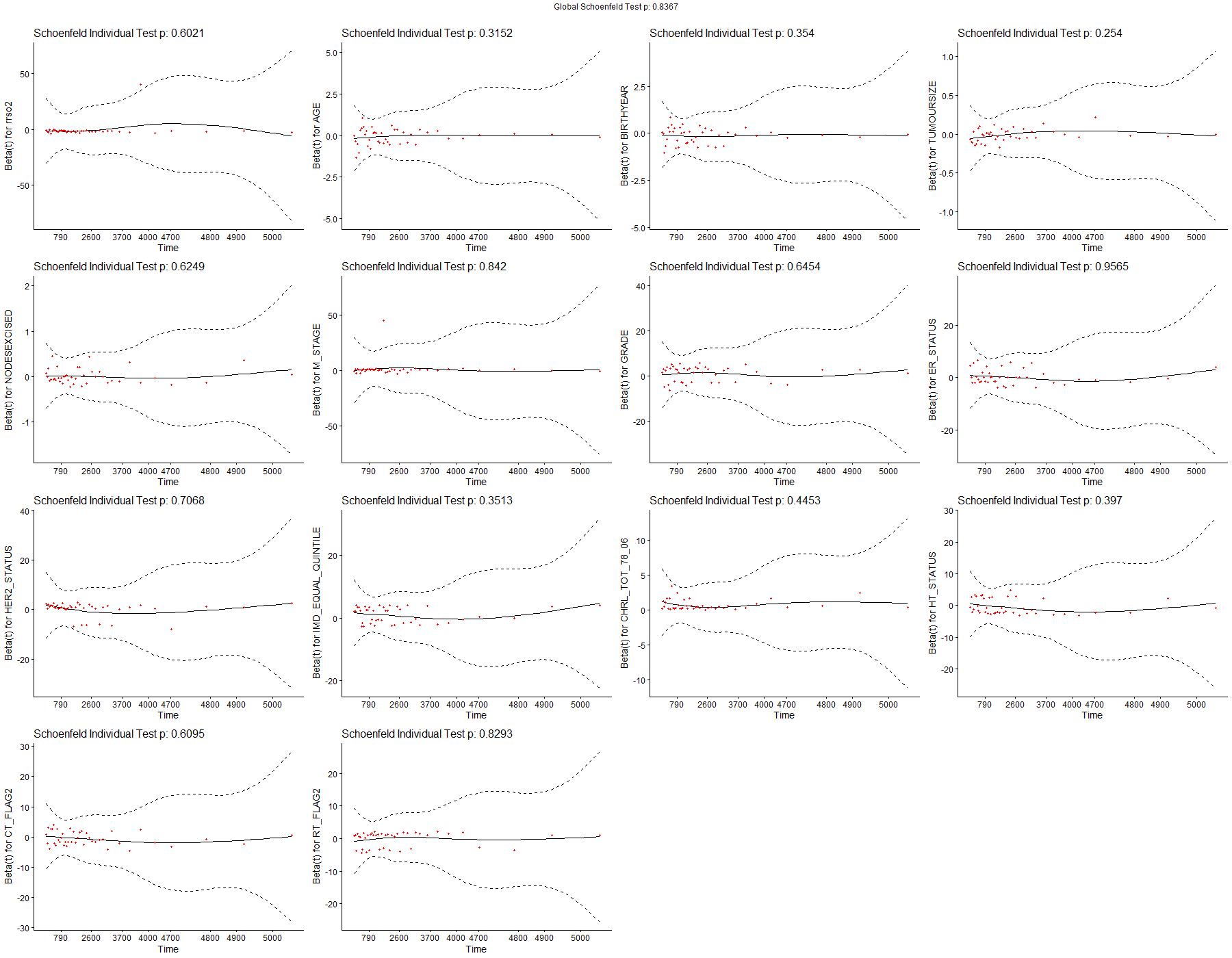


Figure 15S: Depression, BSO ≥55


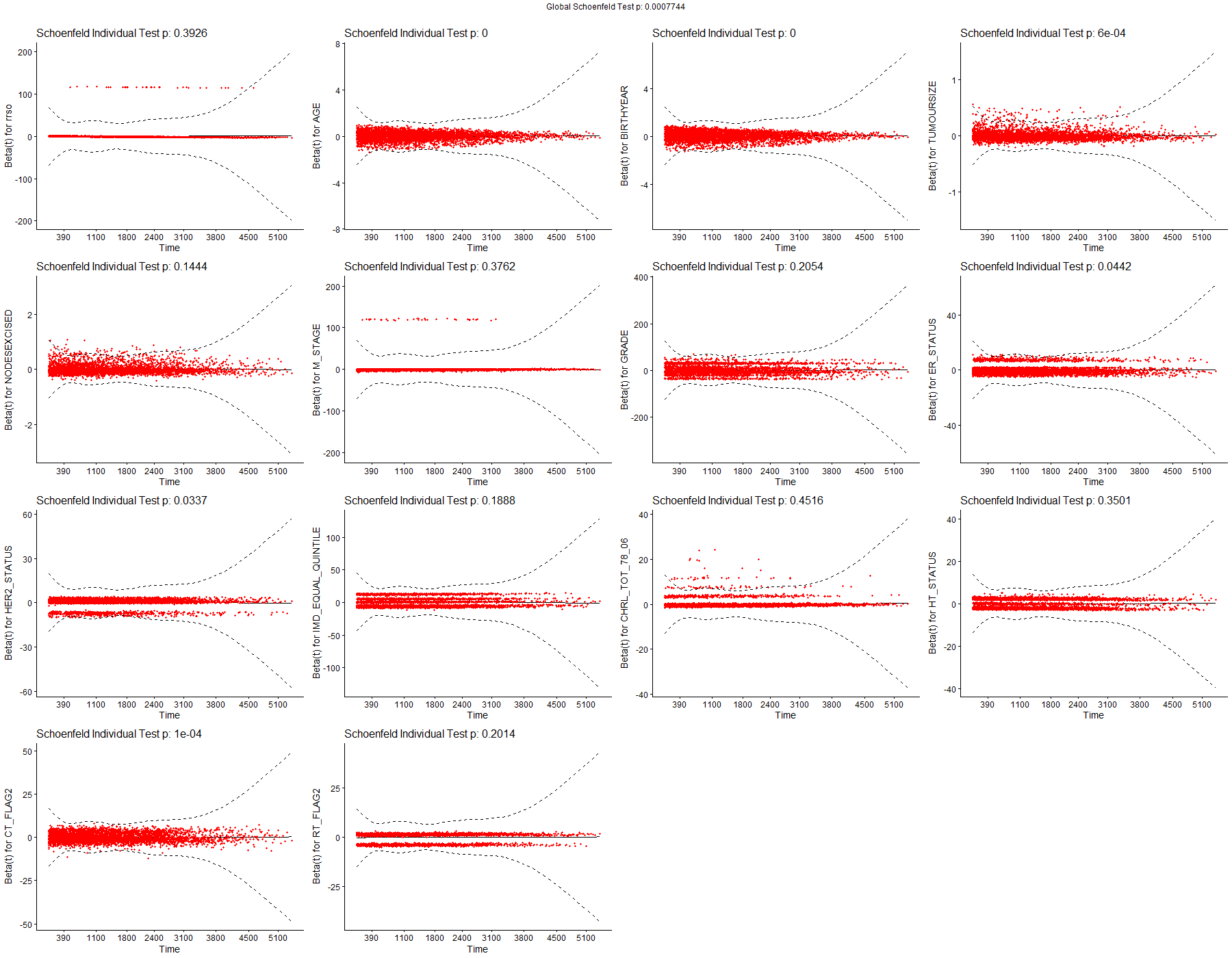


Figure 16S: Depression, BSO<55


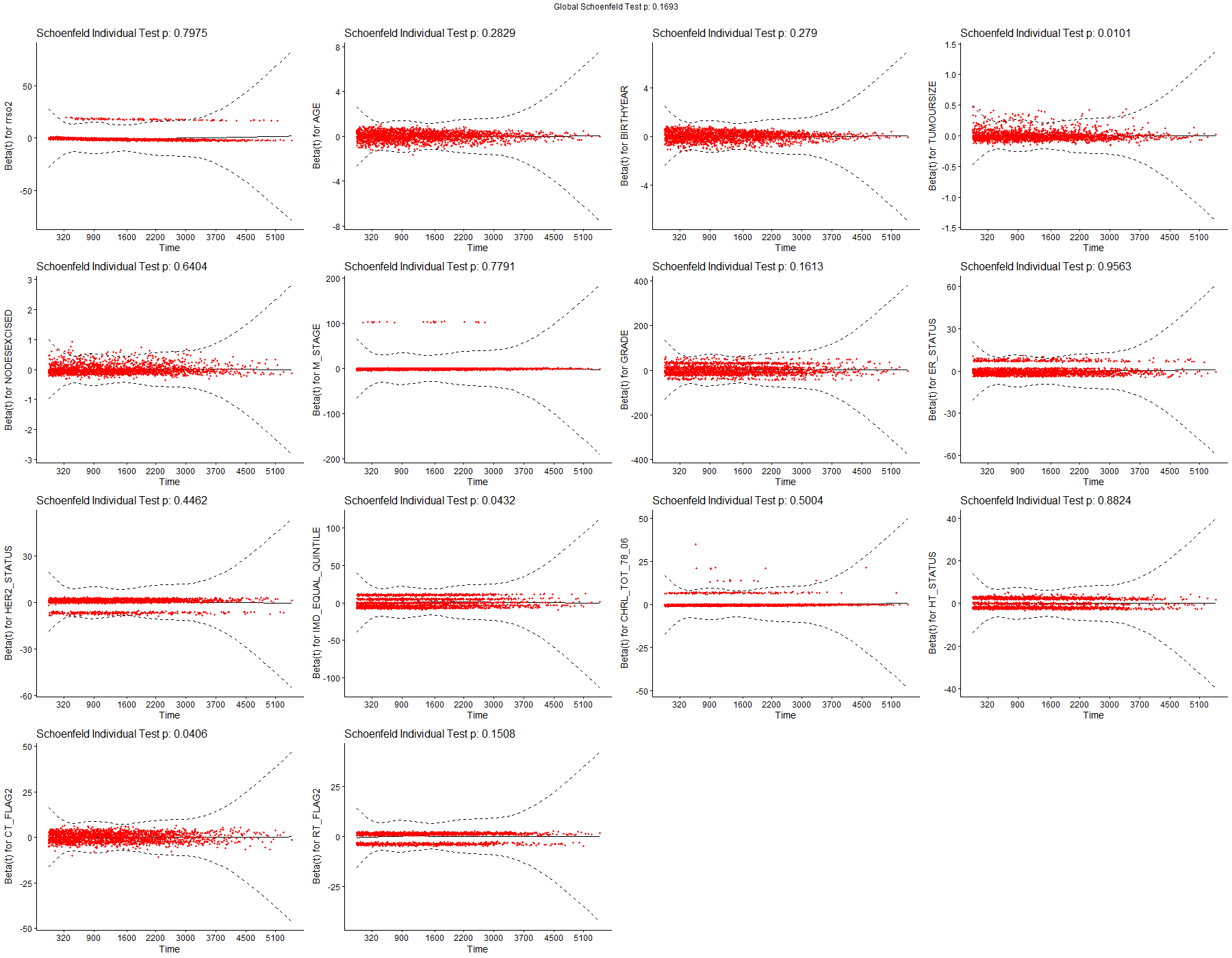


Figure 17S: Parkinsonism, BSO≥55


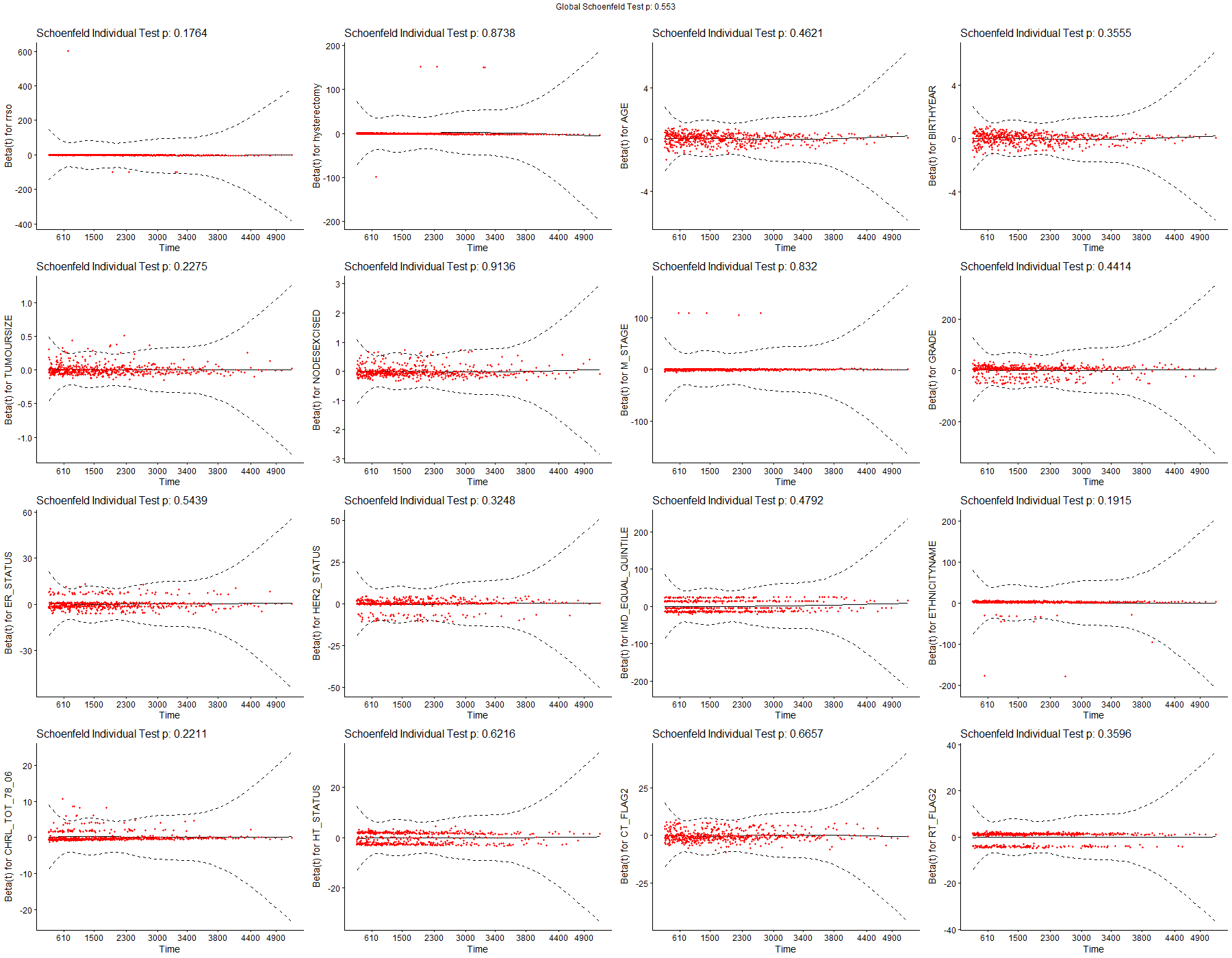


Figure 18S: Parkinsonism, BSO<55


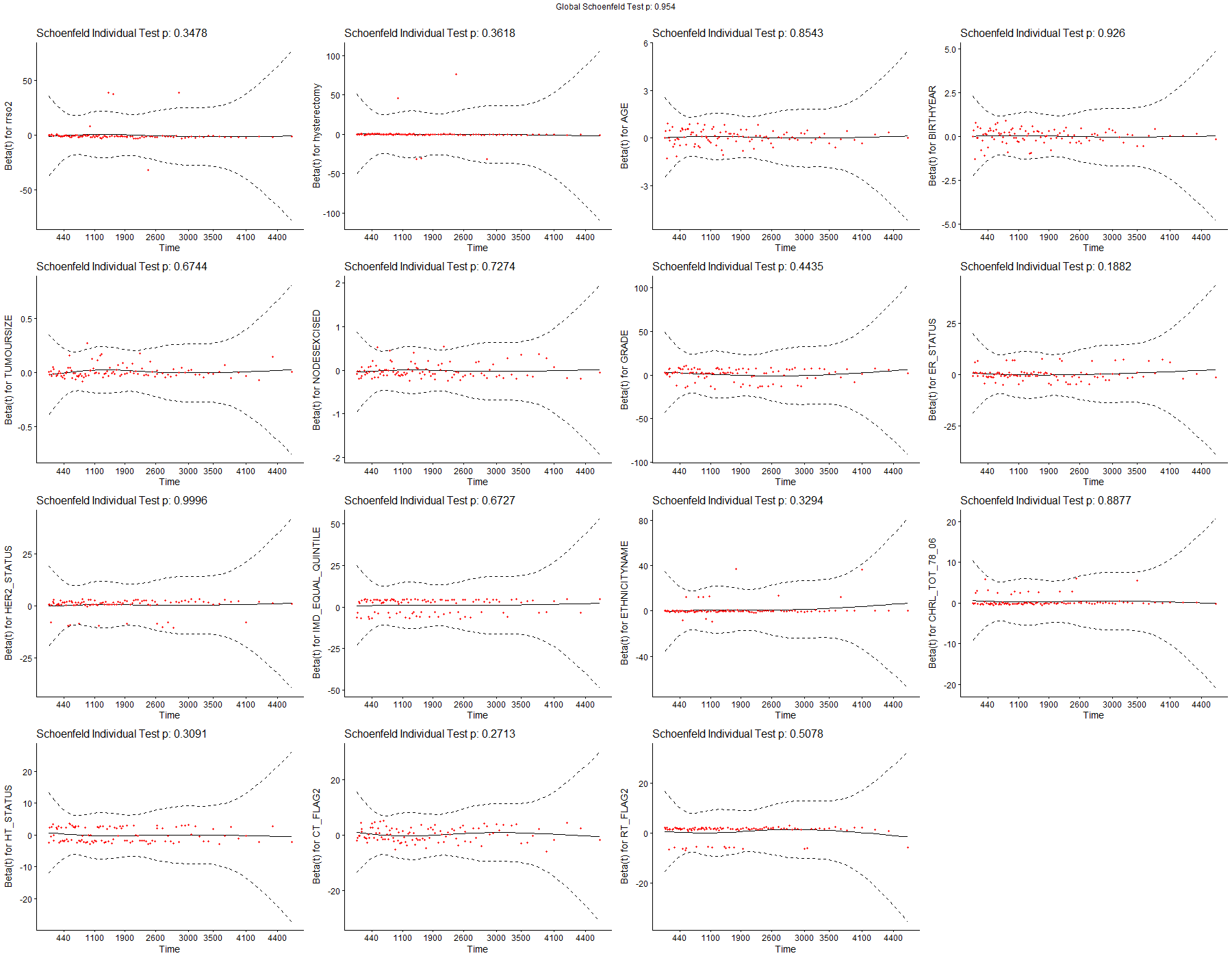


Table7S: Association between BSO before age 55 by indication (prophylactic/other benign) and breast cancer mortality.

| Age at BSO | Indication | HR (95% CI) |
| --- | --- | --- |
| < 55 | Prophylactic indication | 1.10 (1.03-1.17) |
|  | Other benign indication | 1.07 (1.00-1.15) |

# References

1. White, I.R. and P. Royston, *Imputing missing covariate values for the Cox model.* Stat Med, 2009. **28**(15): p. 1982-98.
